# An algebraic characterization of self-generating chemical reaction networks using semigroup models

**DOI:** 10.1007/s00285-023-01899-4

**Published:** 2023-04-18

**Authors:** Dimitri Loutchko

**Affiliations:** Institute of Industrial Science, The University of Tokyo, 4-6-1, Komaba, Meguro-ku, Tokyo, 153-8505 Japan

**Keywords:** Biochemical reaction networks, Autocatalytic sets, Algebraic models, Finite semigroups

## Abstract

The ability of a chemical reaction network to generate itself by catalyzed reactions from constantly present environmental food sources is considered a fundamental property in origin-of-life research. Based on Kaufmann’s autocatalytic sets, Hordijk and Steel have constructed the versatile formalism of catalytic reaction systems (CRS) to model and to analyze such self-generating networks, which they named reflexively autocatalytic and food-generated. Recently, it was established that the subsequent and simultaenous catalytic functions of the chemicals of a CRS give rise to an algebraic structure, termed a *semigroup model*. The semigroup model allows to naturally consider the function of any subset of chemicals on the whole CRS. This gives rise to a generative dynamics by iteratively applying the function of a subset to the externally supplied food set. The fixed point of this dynamics yields the maximal self-generating set of chemicals. Moreover, the set of all functionally closed self-generating sets of chemicals is discussed and a structure theorem for this set is proven. It is also shown that a CRS which contains self-generating sets of chemicals cannot have a nilpotent semigroup model and thus a useful link to the combinatorial theory of finite semigroups is established. The main technical tool introduced and utilized in this work is the representation of the semigroup elements as decorated rooted trees, allowing to translate the generation of chemicals from a given set of resources into the semigroup language.

## Introduction

Questions about the origin of life are as fascinating as they are difficult to even attempt to answer. There are at least two schools of thought on how to approach such questions. The first one is to construct minimal models involving concrete chemicals, best exemplified by the RNA world hypothesis formulated by Gilbert (1986), Joyce (1989) and many others. The great advantage of such concrete models is that they can be tested experimentally, going all the way back to the classical experiments by Miller (1953) and Oró (1961). However, there can never be certainty about any hypothesized model, and even the most convincing ones such as the RNA world hypothesis lack reliable data with regard to their first appearance, cf. Joyce (2002); Penny (2005). An alternative school of thought is focused on working out the minimal requirements which any sensible theory of the origin of life should satisfy. Prominent proponents of this approach are Oparin (1957), Dyson (1999), Kauffman (1986), and many others. However, already the formulation of a meaningful theoretical framework is challenging and there have been various attempts including (*M*, *R*)-systems by Rosen (1958), hypercycles by Eigen (1971), autopoetic systems by Varela et al. (1974), chemotons by Gánti (1975) and autocatalytic sets by Kauffman (1986). A common feature that all frameworks have in common is the importance of autocatalysis and the occurrence of autocatalytic cycles as discussed in the review by Hordijk and Steel (2018).

The catalytic reaction system (CRS) formalism by Steel (2000); Hordijk and Steel (2004) is a versatile framework that, motivated by Kauffman’s autocatalytic sets, captures the essence of several of the aforementioned approaches. It has been used to compute thresholds for the occurrence of self-generating and self-sustaining motives in CRS based on the level of catalysis by Hordijk et al. (2010, 2011, 2012, 2015); Hordijk and Steel (2017, 2018). As reviewed by Steel et al. (2019), the algorithms developed for CRS to find such motives have polynomial runtimes and can therefore be applied to very large networks. They have succesfully been applied, for example by Sousa et al. (2015); Xavier et al. (2020); Xavier and Kauffman (2022) to identify self-generating and self-sustaining motives in CRS which capture the whole metabolism of primitive organisms and consist of thousand of chemicals and reactions. The CRS formalism is even capable of analyzing the structure economic organization, as was demonstrated by Hordijk (2013).

In the companion article by Loutchko (2022), it has been shown that CRS have an algebraic structure that is generated by the simultaneous and subsequent function of chemicals acting as catalysts on the CRS. It was then shown how a naturally defined discrete dynamics yields the maximal self-sustaining set of chemicals for any given CRS and a characterization of the set of functionally closed self-sustaining sets of chemicals was derived. This article aims to achieve the same for self-generating sets of chemicals, which is a stricter notion than that of self-sustainment and requires more mathematical care. In this regard, the main technical contribution of this article is to construct a representation of the semigroup elements as decorated rooted trees as they are naturally suited to deal with the *generation* of chemicals from a set of externally supplied chemicals. Some of the methods developed in this article can be viewed as algebraic reincarnations of the known algorithms for CRS (cf. Steel et al. (2019) for an overview) and thus they should also be applicable to the analysis of very large systems due to their polynomial runtime.

***Mathematical outline***

This section provides a mathematical overview of the article, with an emphasis on the main results. For the sake of brevity and consistency, an example is not provided here but the reader who wishes to build more intuition is referred to the example in the “Appendix” by Loutchko (2022), where the dynamics $Y \mapsto \Phi _Y(Y)$ should be replaced by $Y \mapsto \Phi _Y(\emptyset )$.

The construction of the semigroup models is based on the CRS formalism introduced by Hordijk and Steel (2004); Hordijk et al. (2011). A CRS is given by the datum of a chemical reaction network, i.e. a finite set of chemicals *X* together with a finite set of reactions *R* where each reaction $r \in R$ is determined by the set of its reactants $\text {dom}(r) \subset X$ and products $\text {ran}(r) \subset X$. Additionally, catalysis data is specified by a set $C \subset X \times R$ meaning that for each $(x,r) \in C$, the reaction *r* is catalyzed by the chemical *x*, and a food set $F \subset X$ of constantly supplied chemicals is given. A CRS is said to be RAF (reflexively autocatalytic and food-generated) if each chemical in the CRS can be generated from the food set *F* by a series of catalyzed reactions. A set of chemicals is said to be RAF if the CRS generated by it is RAF. The notion of RAF formalizes self-generating reaction networks in the framework of CRS. Details on CRS are given in Sect. 2.1.

In Sect. 2.2, it is shown that the reactions and the catalytic functions of chemicals have the structure of a semigroup, which is additionally equipped with a partial order and an idempotent addition. The semigroup operation corresponds to subsequent functionality whereas the addition corresponds to simultaneous application of functions. More precisely, to each reaction $r \in R$ a function $\phi _r$ is assigned as the set-map $\phi _r: {\mathfrak {X}} \rightarrow {\mathfrak {X}}$ on the power set ${\mathfrak {X}}:={\mathcal {P}}(X_F)$ of non-food chemicals $X_F = X \setminus F$. The function $\phi _r$ gives the set of non-food products of *r* if the set of non-food reactants of *r* is contained in its argument and $\emptyset$ otherwise. Such functions have the usual composition given by ${(\phi _r \circ \phi _{r'})(Y) = \phi _r (\phi _{r'}(Y))}$ and an idempotent addition given by ${(\phi _r + \phi _{r'})(Y) = \phi _r(Y) \cup \phi _{r'}(Y)}$ for all $Y \subset X_F$ and $r,r' \in R$. They generate the semigroup model

$$\begin{aligned} {\mathcal {S}}^R = \langle \phi _r \rangle _{r \in R}. \end{aligned}$$

To each of the chemicals $x \in X$, a function $\phi _x: {\mathfrak {X}} \rightarrow {\mathfrak {X}}$ is assigned by using the catalysis data:

$$\begin{aligned} \phi _x = \sum _{(x,r) \in C} \phi _r. \end{aligned}$$

The functions of the chemicals generate the semigroup model

$$\begin{aligned} {\mathcal {S}} = \langle \phi _x \rangle _{x \in X}, \end{aligned}$$

which is a subsemigroup of ${\mathcal {S}}^R$. The objects ${\mathcal {S}}^R$ and ${\mathcal {S}}$ are semigroups with respect to both $+$ and $\circ$, hence they are called *semigroup models*.

The elements of the semigroup models are partially ordered via $\phi \le \psi$ iff $\phi (Y) \subset \psi (Y)$ for all $Y \subset X_F$. Lemma 2.13 states that the partial order on the semigroup models, the partial order on ${\mathfrak {X}}$, and the two operations $\circ$ and $+$ are all compatible. A central notion is the function $\Phi _Y \in {\mathcal {S}}$ of a set of non-food chemicals $Y \subset X_F$ which is defined as the unique maximal element of the subsemigroup

$$\begin{aligned} {\mathcal {S}}(Y) = \langle \phi _x \rangle _{x \in Y \cup F} \end{aligned}$$

of ${\mathcal {S}}$. The function $\Phi _Y$ captures all catalytic functionality that can be exerted by *Y* and the food set on all other chemicals of the CRS.

**Fig. 1:**
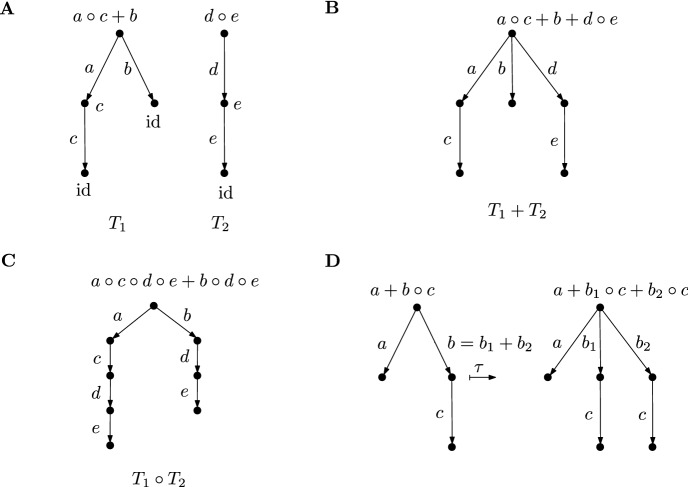
An illustration of the algebra of decorated rooted trees. **A** The edge labels $a,b,c,d,e \in A$ determine the vertex labels of the two trees $T_1$ and $T_2$ recursively: the leaves are labelled by the multiplicatively neutral element and each vertex function is given by the summation of the labels over all outgoing edges, multiplied with the labels of the vertices at their heads. All edge and the resulting vertex labels are shown here, whereas in B,C and D only the labels of the edges and of the root are shown. **B** Addition of the two trees $T_1$ and $T_2$: The roots of both trees are identified and the labels on all edges of both trees are retained. The vertex labels are determined as in A. The root label of $T_1 + T_2$ is equal to the sum of the root labels of $T_1$ and $T_2$. **C** Multiplication of two trees $T_1$ and $T_2$: Each leaf of $T_1$ is replaced with a copy of $T_2$. Thereby, the edge labels from the original trees are retained, which yields the respective vertex labels. If the right distributivity of the operations $+$ and $\circ$ holds, then the root label of $T_1 \circ T_2$ is equal to the concatenation of the root labels of $T_1$ and $T_2$. **D** The replacement of an edge with label $b = b_1 + b_2$ by two edges with labels $b_1$ and $b_2$. A copy of the child tree of the original edge is attached to each of the new edges. If the right distributivity of the operations $+$ and $\circ$ holds, then the root labels of both shown trees are equal

Section 3 provides more insight into the structure of the semigroup models. The basis is the definition of a tree algebra ${\mathfrak {T}}(A)$ with a decorating algebra $(A,\circ ,+)$ as follows: The objects in ${\mathfrak {T}}(A)$ are rooted trees, whose edge labels are arbitrary elements in *A*. The vertrex labels are determined by these edge labels: All leaves are labelled by the multiplicatively neutral element $\text {id}$. At each non-leaf vertex the labels of the outgoing edges are multiplied with the labels on the respective child vertex and the sum is taken over all the outgoing edges. This is illustrated in Fig. 1A. The addition of trees is performed by identifying their roots, with unchanged labels at the edges, as illustrated in Fig. 1B. The multiplication of trees $T_1 \circ T_2$ is carried out by replacing all leaves of $T_1$ with copies of $T_2$. Again, all edge labels are unchanged, as illustrated in Fig. 1C.

The tree algebras relevant for semigroup models have their edges labelled by the generating sets of the respective models, i.e. they are ${{\mathfrak {T}} := {\mathfrak {T}}(\{\phi _x\}_{x \in X} \cup \{0\})}$ and ${{\mathfrak {T}}^R := {\mathfrak {T}}(\{\phi _r\}_{r \in R} \cup \{0\})}$. The main result of the section is Theorem 3.8, which states that there is a commutative diagram of homomorphisms 
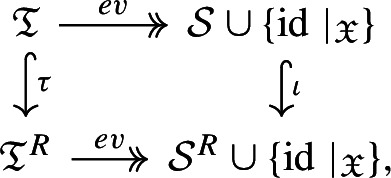


whereby the surjective evaluation map *ev* sends the root label to the corresponding semigroup element and the map $\tau$ is defined based on the formula $\phi _x = \sum _{(x,r) \in C} \phi _r$. More precisely, $\tau$ replaces an edge with the label $\phi _x$ by edges labeled by $\phi _r$ for each $(x,r) \in C$ and a copy of the child tree of the original edge is attached to each of the new edges, as illustrated in Fig. 1D. A tree representing a semigroup element is a lift of the element via the evaluation homomorphism *ev*. The algebraic reason for the existence of such representations is the interplay of the two operations $\circ$ and $+$ via the right distributivity $\phi \circ \chi + \psi \circ \chi = (\phi + \psi ) \circ \chi$ for any $\phi , \psi , \chi \in {\mathcal {S}}^R$.

Loosely speaking, the trees in ${\mathfrak {T}}^R$ correspond to “reaction mechanisms”, which proceed recursively from the leaves to the root such that a reaction labeling an edge occurs subsequently with the “mechanism” of its head vertex and such that all reactions labeling edges with the same tail are carried out simultaneously. Thus, it is natural to assume that a chemical $x \in X_F$ can be generated from the food set if there is a reaction mechanism for its generation, given by a tree $T \in {\mathfrak {T}}^R$. This translates to $x \in ev(T)(\emptyset )$ in this setup. And indeed, it is proven in Lemma 3.13 that this property is equivalent to the standard definition of generation from the food set.

In Sect. 4, it is shown how the representation of semigroup elements by decorated rooted trees can be used to describe CRS with the RAF property by the simple condition $\Phi _{X_F}(\emptyset ) = X_F$ (Theorem 4.1). This implies that for a RAF set of chemicals $X'_F \subset X_F$, the property $X_F' \subset \Phi _{X_F'}(\emptyset )$ holds (Corollary 4.2) and, moreover, that the equality $X_F' = \Phi _{X_F'}(\emptyset )$ is a sufficient condition for $X'_F$ to be a RAF set of chemicals (Proposition 4.3). Then, a generative dynamics on ${\mathfrak {X}}$ is defined by $Y \mapsto \Phi _Y(\emptyset )$, and the fixed point of the dynamics with initial condition $Y_0$ is denoted by $Y_0^{*g}$ (when it exists). As one of the main results, it is proven that the dynamics with initial condition given by $X_F$ leads to the maximal RAF set of chemicals.

Finally, new insights and conjectures gained from the semigroup approach to CRS are discussed. If a set $Y_0 \subset X_F$ satisfies $Y_0 \subset \Phi _{Y_0}(\emptyset )$, then it is not stable but will produce all chemicals in $Y_1 := \Phi _{Y_0}(\emptyset )$ over time. This process continues until the fixed point $Y_0^{*g}$ is reached. The set $Y_0^{*g}$ does not produce any further chemicals and satisfies $Y_0^{*g} = \Phi _{Y_0^{*g}}(\emptyset )$. The sets which satisfy this fixed point equation are called *functionally closed RAF sets* of chemicals, and they are a subclass of self-generating sets according to Theorem 4.9. Their precursors, i.e., sets which satisfy $Y \subset \Phi _{Y}(\emptyset )$, are termed *pre-functionally closed RAF sets* of chemicals, and they contain all self-generating sets of chemicals by Corollary 4.2. A characterization of the set of all pre-functionally closed RAF sets of a CRS is provided in Theorem 4.18. It is based on the reduced generative dynamics given by $Y \mapsto Y \cap \Phi _Y(\emptyset )$. This dynamics always has a fixed point, denoted by $Y_0^{*rg}$ for the inital condition $Y_0$. For each set $Y \subset X_F$, the set of fixed points

$$\begin{aligned} {\mathfrak {p}}(Y) := \left\{ (Y \setminus \{y\})^{*rg} \text { for }y \in Y \right\} \end{aligned}$$

is introduced and one recursively defines

$$\begin{aligned} \mathfrak {pF}^0&:= \{ X_F^{*g} \} \\ \mathfrak {pF}^{i+1}&:= \bigcup _{Y \in \mathfrak {pF}^i} {\mathfrak {p}}(Y) \text { for all }i \in {\mathbb {Z}}_{\ge 0}. \end{aligned}$$

The statement of the Theorem 4.18 is that the set of pre-functionally closed RAF sets of chemicals is given by

$$\begin{aligned} \mathfrak {pF} := \bigcup _{i=0}^{\mid X_F^{*g} \mid } \mathfrak {pF}^i. \end{aligned}$$

The set ${\mathfrak {F}}$ of all functionally closed RAF sets is obtained from $\mathfrak {pF}$ as

$$\begin{aligned} {\mathfrak {F}} = \{ Y^{*g} \text { for } Y \in \mathfrak {pF} \}, \end{aligned}$$

which is proven in Lemma 4.16.

In the concluding Sect. 5, the importance of the representations of semigroup elements by decorated rooted trees is discussed, and the biochemical significance of functionally closed RAF sets of chemicals is illustrated. For example, one would expect chemicals which are uniquely contained in a minimal functionally closed RAF set of chemicals to be involved solely in the functionality of the respective RAF set, whereas chemicals that have multiple minimal functionally closed RAF sets of chemicals containing them are more likely to be involved in communication and interaction between the respective RAF sets. This can potentially carry information on the evolutionary role of the respective chemicals. This is an illustration of how the semigroup models can be used to discover new concepts in CRS theory. In future work, such concepts will be applied to CRS corresponding to real biological systems.

## Semigroup models

The construction of semigroup models and their elementary properties are provided in Sect. 2.2. They are based on the catalytic reaction system (CRS) formalism, which is introduced in Sect. 2.1. This is a condensed version of the Sections 2. and 3. from the introductory companion article by Loutchko (2022). Only the RAF property (Definition 2.5) and the extended semigroup model ${\mathcal {S}}^R$ (Definition 2.10) are newly introduced here.

### The CRS formalism

The introduction of the catalytic reaction system (CRS) formalism and of the reflexievly-autocatalytic and food-generated (RAF) property are based on the work of Hordijk and Steel (2004).

The notion of CRS is designed to capture the catalytic functionality within a given chemical reaction network. It does not take into account detailed kinetic or thermodynamic information.

#### Definition 2.1

A *catalytic reaction system* (CRS) is a tuple (*X*, *R*, *C*, *F*) where *X* is a finite discrete set of chemicals, *R* is a finite set of reactions, $C \subset X \times R$ is the catalysis data for the reactions *R* and $F \subset X$ is the constantly present food set.

Each reaction $r \in R$ is given by a pair $(\text {dom}(r),\text {ran}(r))$ of mutually disjoint subsets of *X*, called the *domain* and the *range* of *r*. The elements of $\text {dom}(r)$ are called the reactants and the elements of $\text {ran}(r)$ are the products of *r*. For a pair $(x,r) \in C$, the reaction *r* is said to be *catalyzed* by *x* and *x* is said to be a *catalyst* of *r*. The food set *F* is required to satisfy the following closure property:

(C) All reactions $r \in R$ with a catalyst in *F* must involve chemicals outside of *F* as reactants, i.e. they must satisfy $\text {dom}(r) \cap (X \setminus F) \ne \emptyset$.

If $X=F$, the CRS is said to be *trivial*.

#### Example 2.2

**Fig. 2:**
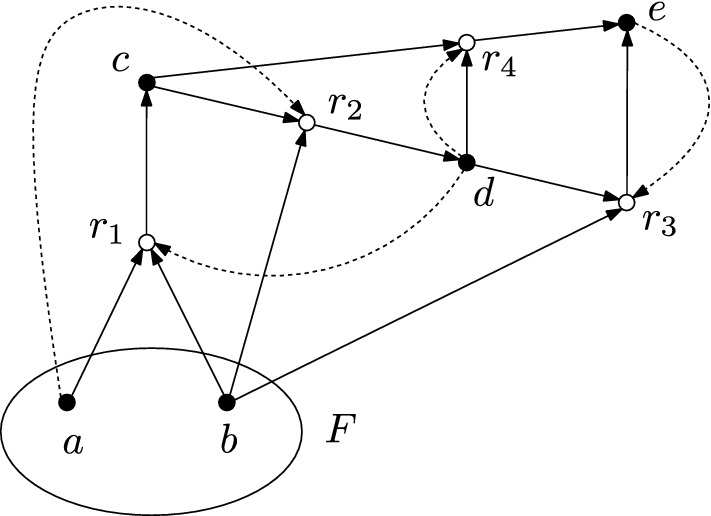
Example of a graphical representation of a CRS. The CRS consists of five chemicals $X=\{a,b,c,d,e\}$ and four reactions $a+b\rightarrow c$, $c+b \rightarrow d$, $b+d \rightarrow e$ and $c+d \rightarrow e$, which are catalyzed by *d*, *a*, *e* and *d*, respectively. The food set is given by $F = \{a,b\}$

Figure 2 shows a representation of a CRS as a directed bipartite graph. This representation is used throughout this article. The chemicals are represented by solid vertices and the reactions $r = (\text {dom}(r),\text {ran}(r))$ are represented by circles. For each reaction, there are directed edges from each chemical in $\text {dom}(r)$ to the reaction vertex and from the reaction vertex to each chemical in $\text {ran}(r)$. The catalysis data $(x,r) \in C$ is indicated by a dashed directed edge from the chemical *x* to the reaction *r*. The food set is indicated by a circle around the food chemicals.

The projection

$$\begin{aligned} \pi _R&: C \rightarrow R, \\&(x,r) \mapsto r \end{aligned}$$

yields the set of all catalyzed reactions of the CRS as $\pi _R(C)$.

The *support* of a reaction *r* is defined as

$$\begin{aligned} \text {supp}(r) := \text {dom}(r) \cup \text {ran}(r) \end{aligned}$$

and the notions of domain, range, and support extend to sets of reactions $R' \subset R$ via

$$\begin{aligned} \text {dom}(R') = \bigcup _{r \in R'} \text {dom}(r) \end{aligned}$$

with the analogous definitions for $\text {ran}(R')$ and $\text {supp}(R')$.

From now on, a CRS (*X*, *R*, *C*, *F*) will be fixed. When referring to any of the four sets *X*, *R*, *C* or *F*, it is implicitly assumed that they are part of the full data of the CRS. It will be convenient to abbreviate the non-food chemicals as

$$\begin{aligned} X_F := X \setminus F \end{aligned}$$

and to make the same definition for any subset $X'$ of *X* containing *F*, i.e. $X'_F := X' \setminus F$. Moreover, given a set $X'_F \subset X_F$, the symbol $X'$ will denote the set $X'_F \cup F \subset X$.

#### Definition 2.3

For a set $X'_F \subset X_F$ of non-food chemicals, define the restrictions of *R* and *C* as

$$\begin{aligned} R\mid _{X'}&= \{ r \in R \text { such that supp}(r) \subset X'\}, \\ C\mid _{X'}&= C \cap (X' \times R\mid _{X'}). \end{aligned}$$

The tuple $(X',R\mid _{X'},C\mid _{X'},F)$ is called the *subCRS generated by* $X'_F$.

In the article by Loutchko (2022), a broader notion of subCRS is introduced. This notion is, however, not needed in this work as the focus will be exclusively on subCRS generated by sets of non-food chemicals. Note that the subCRS according to Definition 2.3 is always closed in the terminology used by Hordijk and Steel (2017), i.e. all reactions of the full CRS with support on $X'$ are actually contained in the respective subCRS.

Now the central notions of a food-generated CRS and a reflexively autocatalytic and food-generated (RAF) CRS are introduced following Hordijk and Steel (2004, 2017). However, the definitions given by Hordijk and Steel (2004, 2017) are centered around the set of reactions *R*, whereas the definitions given here involve the whole CRS. In Remark 2.9, the relation to the definitions used in this work is discussed. The F property formalizes the idea that all chemicals of the CRS can be *generated* from the food set. The RAF property means that the generation from the food set can be achieved with catalyzed reactions only.

#### Definition 2.4

A CRS (*X*, *R*, *C*, *F*) has the *food generation property* (F property) if each $x \in X_F$ is generated by some sequence of reactions from *F*, i.e. if the following condition is satisfied for each $x \in X_F$:

(F) There exists a family of sets of reactions $R_1,...,R_n \subset R$ with the following properties:
  1. $$\text {dom}(R_1) \subset F$$.
  2. $$\text {dom}(R_{i+1}) \subset \bigcup _{j=1}^i\text {ran}(R_j) \cup F$$ for all $$1 \le i \le n-1$$.
  3. $$x \in \text {ran}(R_n)$$.

#### Definition 2.5

A CRS (*X*, *R*, *C*, *F*) is *reflexively autocatalytic and food-generated* (RAF) if it is has the F property and if for each chemical $x \in X_F$, the sets of reactions $R_1,\dots R_n \subset R$ featured in the condition (F) can be chosen to be subsets of $\pi _R(C)$. In other words, the reactions in $R_1,\dots R_n$ are all required to be catalyzed. A CRS which is RAF is also called a *self-generating* CRS.

#### Remark 2.6

**Fig. 3:**
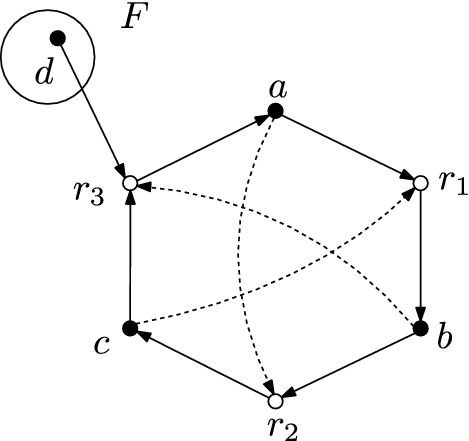
A self-sustaining but not self-generating CRS. The self-sustainment properties of this CRS are discussed in Example 2.7 by Loutchko (2022). The self-generation property fails because the condition (F1) of the food generation property is not satisfied, i.e., there are no reactions $r \in R$ with $\text {dom}(r) \subset F$

The notion of *self-generation* is stronger than the one of *self-sustainment*. Self-sustaining CRS are treated within the semigroup formalism by Loutchko (2022). Self-sustainment requires the CRS to have a catalyzed set of reactions $R' \subset \pi _R(C)$ such that $X_F \subset \text {ran}(R')$. The RAF condition is stronger than this, because one can set $R'_x := \cup _{i=1}^n R_n$ for the reactions featured in condition (F) and $R' = \cup _{x \in X_F} R'_x$ will satisfy the requirement for self-sustainment. On the contrary, there are self-sustaining CRS which are not self-generating. One example is shown in Fig. 3.

The definition of the RAF property descends to sets of non-food chemicals $X'_F \subset X_F$ based on the Definition 2.3.

#### Definition 2.7

A set of chemicals $X'_F \subset X_F$ is said to be a *RAF set of chemicals* or *self-generating set of chemicals* if the subCRS ${(X',R\mid _{X'},C\mid _{X'},F)}$ generated by it is RAF.

#### Example 2.8

The CRS in Fig. 2 is RAF and thus $X_F = \{c,d,e\}$ is a RAF set of chemicals. Moreover, there is a RAF subset of chemicals consisting of $X_F' = \{c,d\}$, because *d* catalyzes the formation of *c* from the food set and *c* reacts with the food set to form *d*, which is catalyzed by the food set.

#### Remark 2.9

(Relation to the notion of RAF commonly used in the literature). The Definition 2.4 of the F property given here coincides *verbatim* with the one commonly used in the CRS literature. The Definition 2.5 of the RAF property is equivalent to the definitions of a closed^1^ RAF set of reactions given by Hordijk and Steel (2004, 2017) modulo the inclusion of uncatalyzed reactions in the set of reactions *R* in the definition given here. Hordijk and Steel (2004, 2017) define the RAF property for subsets of *R* as follows: A subset $R' \subset R$ is a RAF set of reactions if it has the F property^2^ and if each reaction $r \in R'$ is catalyzed by a chemical $x \in \text {supp}(R') \cup F$. Thus, a subCRS $(X',R\mid _{X'},C\mid _{X'},F)$ corresponds to the RAF set of reactions $\pi _{R'}(C')$ and, *vice versa*, a closed RAF set of reactions $R'$ corresponds to the CRS $(X',R\mid _{X'},C\mid _{X'},F)$ with $X' := \text {supp}(R')$.

One can easily lift the restriction of the RAF sets of reactions being closed by defining subCRS with sets of chemicals $X'$ to allow for arbitrary sets of reactions $R' \subset R\mid _{X'}$. This construction is given by Loutchko (2022).

### The semigroup model of a CRS

The chemical reactions of a CRS have a natural algebraic structure given by the simultaneous and subsequent occurrence of reactions, as well as combinations thereof. Making this mathematically precise leads to the notion of an extended semigroup model ${\mathcal {S}}^R$ of a CRS. The function of a chemical is defined by the simultaneous occurrence of all the reactions it catalyzes. All combinations of subsequent and simultaneous functions of chemicals give rise to the semigroup model ${\mathcal {S}}$ of a CRS. The construction of the semigroup models is motivated by the work of Rhodes and Nehaniv (2010) in spirit, but technically the objects constructed here differ significantly, cf. Loutchko (2022), Remark 3.4.

Throughout this section, let (*X*, *R*, *C*, *F*) be a CRS. The state of the CRS is defined by the presence or absence of each of the non-food chemicals, i.e. by a subset $Y \subset X_F$. Therefore, the state space ${\mathfrak {X}}$ of the CRS is the power set

$$\begin{aligned} {\mathfrak {X}} := {\mathcal {P}}(X_F) = \{0,1\}^{X_F}. \end{aligned}$$

A reaction $r \in R$ acts on the state space via its *function*

$$\begin{aligned} \phi _r : {\mathfrak {X}} \rightarrow {\mathfrak {X}} \end{aligned}$$

given by

2.1 $$\begin{aligned} \phi _r(Y) ={\left\{ \begin{array}{ll} \text {ran}(r) \cap X_F &{} \text {if}\, \, \text {dom}(r) \subset Y \cup F\\ \emptyset &{} \text {else} \end{array}\right. } \end{aligned}$$

for all $Y \subset X_F$. Two maps $\phi , \psi : {\mathfrak {X}} \rightarrow {\mathfrak {X}}$ can be composed via the addition $+$, which is defined as

2.2 $$\begin{aligned} (\phi + \psi )(Y) = \phi (Y) \cup \psi (Y) \end{aligned}$$

for all $Y \subset X_F$. This operation is associative, commutative and idempotent. Moreover, the multiplication $\circ$ is given by the usual composition of maps

2.3 $$\begin{aligned} (\phi \circ \psi )(Y) := \phi (\psi (Y)) \end{aligned}$$

for all $$Y \subset X_F$$.

Finally, the *function* $\phi _x: {\mathfrak {X}} \rightarrow {\mathfrak {X}}$ of a chemical $x \in X$ is defined as the sum over all reactions catalyzed by it via

2.4 $$\begin{aligned} \phi _x = \sum _{(x,r) \in C} \phi _r. \end{aligned}$$

Recall that the *full transformation semigroup* ${\mathcal {T}}(A)$ of a finite discrete set *A* is the set of all maps $\{f:A \rightarrow A\}$, where the semigroup operation $\circ$ is the composition of maps. The semigroup model of a CRS is a subsemigroup of ${\mathcal {T}}({\mathfrak {X}})$ and is defined as follows.

#### Definition 2.10

The *semigroup model* ${\mathcal {S}}$ of a CRS is a subsemigroup of ${\mathcal {T}}({\mathfrak {X}})$ generated by the functions $\{\phi _x\}_{x \in X}$ through the operations of addition and composition, i.e. ${\mathcal {S}}$ is the smallest subsemigroup of the full transformation semigroup ${\mathcal {T}}({\mathfrak {X}})$ closed under $\circ$ and $+$ that contains $\{\phi _x\}_{x \in X}$ and the zero function, given by $0(Y) = \emptyset$ for all $Y \subset X_F$. It is denoted by

$$\begin{aligned} {\mathcal {S}} = \langle \phi _x \rangle _{x \in X}. \end{aligned}$$

Analogously, the *extended semigroup model* of the CRS is generated by the functions $\phi _r$ of all reactions $r \in R$. This model is denoted as

$$\begin{aligned} {\mathcal {S}}^R = \langle \phi _r \rangle _{r \in R}. \end{aligned}$$

As subsemigroups of ${\mathcal {T}}({\mathfrak {X}})$, the semigroups ${\mathcal {S}}$ and ${\mathcal {S}}^R$ are *finite*. The objects ${\mathcal {S}}$ and ${\mathcal {S}}^R$ are called semigroup models, because they are semigroups with respect to both operations $\circ$ and $+$. The correct description in terms of universal algebra is, however, an algebra of type (2, 2, 0), cf. Almeida (1995). The semigroup model ${\mathcal {S}}^R$ contains ${\mathcal {S}}$ as a subalgebra of type (2, 2, 0) and this will be expressed by saying that ${\mathcal {S}}$ is a *subsemigroup model* of ${\mathcal {S}}^R$.

#### Remark 2.11

In addition to the two algebraic operations, there is a natural partial order on ${\mathcal {S}}^R$ and ${\mathcal {S}}$, given by $\phi \le \psi \Leftrightarrow \phi (Y) \subset \psi (Y)$ for all $Y \subset X_F$ for $\phi , \psi \in {\mathcal {S}}^R$.

There is an important subsemigroup of ${\mathcal {S}}$ generated by the functions of chemicals in a given set $X_F' \subset X_F$ together with the food set:

#### Definition 2.12

For a subset $X'_F$ of $X_F$, the semigroup model ${\mathcal {S}}(X_F')$ generated by the functions of $X_F'$ is defined as

$$\begin{aligned} {\mathcal {S}}(X'_F) = \langle \phi _x \rangle _{x \in X'} \end{aligned}$$

and the *function* $\Phi _{X_F'}$ of the set $X_F'$ is given by

$$\begin{aligned} \Phi _{X_F'} = \sum _{\phi \in {\mathcal {S}}(X_F')} \phi . \end{aligned}$$

By definition, ${\mathcal {S}}(X_F')$ is a subsemigroup model of ${\mathcal {S}}$. The semigroup models satisfy the following elementary properties. These properties follow directly from the definitions. However, if necessary, the proofs for the respective statements on ${\mathcal {S}}$ can be found in Loutchko (2022), Section 3.2., and the proofs for ${\mathcal {S}}^R$ are analogous.

#### Lemma 2.13

(Elementary properties of semigroup models)

1. All elements $\phi \in {\mathcal {S}}^R$ respect the partial order on ${\mathfrak {X}}$ given by inclusion of sets, i.e.
  $$\begin{aligned} Z \subset Y \subset X_F \implies \phi (Z) \subset \phi (Y). \end{aligned}$$
2. The partial order is compatible with addition and multiplication, i.e. for any $\phi , \phi ', \psi , \psi ' \in {\mathcal {S}}^R$ the following relations hold
  2.5 $$\begin{aligned} \phi \le \psi \text { and } \phi ' \le \psi '&\Rightarrow \phi \circ \phi ' \le \psi \circ \psi ', \end{aligned}$$
  2.6 $$\begin{aligned} \phi \le \psi \text { and } \phi ' \le \psi '&\Rightarrow \phi + \phi ' \le \psi + \psi '. \end{aligned}$$
3. Any $$\phi , \psi \in {\mathcal {S}}$$ satisfy
  $$\begin{aligned} \phi \le \phi + \psi . \end{aligned}$$
4. Any $\phi , \phi ', \psi \in {\mathcal {S}}$ such that $\phi \le \psi$ and $\phi ' \le \psi$ satisfy
  $$\begin{aligned} \phi + \phi ' \le \psi . \end{aligned}$$
5. The operations $\circ$ and $+$ on ${\mathcal {S}}^R$ have the following distributivity properties:
  2.7 $$\begin{aligned} \phi \circ \chi + \psi \circ \chi&= (\phi + \psi ) \circ \chi , \end{aligned}$$
  2.8 $$\begin{aligned} \chi \circ \phi + \chi \circ \psi&\le \chi \circ (\phi + \psi ) \end{aligned}$$
  hold for any $\phi , \psi , \chi \in {\mathcal {S}}^R$.
6. The right distributivity in Equation (2.7) holds more generally for arbitrary elements $\phi , \psi , \chi \in {\mathcal {T}}({\mathfrak {X}})$.
7. $\Phi _{X'_F}$ is the unique maximal element of ${\mathcal {S}}(X'_F)$. In particular, ${\mathcal {S}}$ has a unique maximal element, given by $\Phi _{X_F}$.
8. The functions of sets $X''_F \subset X'_F \subset X_F$ satisfy
  $$\begin{aligned} \Phi _{X''_F} \le \Phi _{X'_F}. \end{aligned}$$

#### Remark 2.14

Any subCRS $(X',R\mid _{X'},C\mid _{X'},F)$ generated by the set of chemicals $X_F'$ has a semigroup model given by Definition 2.10, which will be denoted by ${\mathcal {S}}'(X',R{\mid }_{X'})$. It is a subsemigroup of the full transformation semigroup ${\mathcal {T}}({\mathcal {P}}(X'_F))$ on the power set of $X'_F$. Any element $\phi \in {\mathcal {S}}'(X',R\mid _{X'})$ can be extended to a function $ext(\phi ) \in {\mathcal {T}}({\mathfrak {X}})$ via

$$\begin{aligned} ext(\phi )(Y) = \phi (Y \cap X'_F) \end{aligned}$$

for $Y \subset X_F$. By definition, the generators $\{\phi '_x\}_{x \in X'} \subset {\mathcal {T}}({\mathcal {P}}(X'_F))$ of ${\mathcal {S}}'(X',R\mid _{X'})$ and the generators $\{\phi _x\}_{x \in X'} \subset {\mathcal {T}}({\mathfrak {X}})$ of ${\mathcal {S}}(X'_F)$ satisfy ${ext(\phi '_x) \le \phi _x}$ for all $x \in X'$. Together with the property (S2) this yields the inequality

2.9 $$\begin{aligned} ext(\Phi '_{X'_F}) \le \Phi _{X'_F} \end{aligned}$$

for the maximal functions $\Phi '_{X'_F}$ and $\Phi _{X'_F}$ of ${\mathcal {S}}'(X',R\mid _{X'})$ and ${\mathcal {S}}(X'_F)$.

This finishes the summary of the elementary properties of the semigroup models. In the next section, a representation of the semigroup elements, which is well suited to deal with the condition (F) in food-generated CRS, is constructed.

## Semigroup models as algebras of decorated rooted trees

This section is dedicated to the construction of a representation of elements of ${\mathcal {S}}$ as decorated rooted trees. It forms the technical basis for the proofs in the next section. Albeit the main idea of this section is rather straightforward, the verification of all the claimed properties requires some care. Therefore, the reader might prefer to skip this section up until Theorem 3.8 during the first reading.

**Fig. 4:**
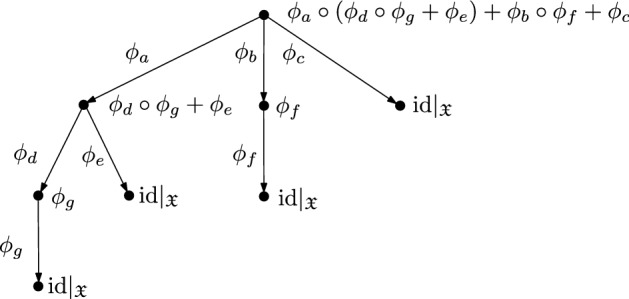
Example of a decorated rooted tree with decorations from the generating set $A = \{ \phi _x \}_{x \in X_F} \cup \{0\}$ of a semigroup model ${\mathcal {S}} = \langle \phi _x \rangle _{x \in X_F} \subset {\mathcal {T}}({\mathfrak {X}})$. The labels of the edges determine the labels on the vertices recursively: At each vertex, a sum over the labels of its children, multiplied by the labels on the respective connecting edges, is taken. The edges are labeled to the left of the respective edge and the resulting labels of the vertices are on the right of the respective vertex. The root is labelled by the function $\phi _a \circ (\phi _d \circ \phi _g + \phi _e) + \phi _b \circ \phi _f + \phi _c \in {\mathcal {S}}$

**Fig. 5:**
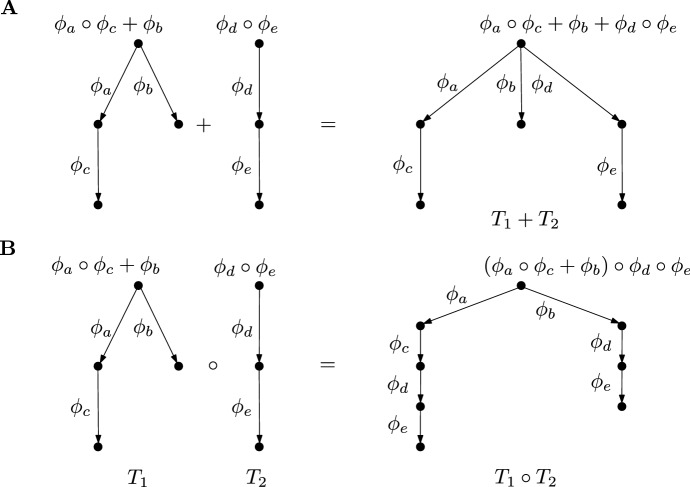
**A** Addition of two trees $T_1$ and $T_2$: The roots of both trees are identified and the labels on all edges of both trees are retained. All vertex labels are given by the relation (3.1). **B** Multiplication of two trees $T_1$ and $T_2$: Each leaf of $T_1$ is replaced with a copy of $T_2$. Thereby the edge labels from the original trees are retained, yielding the vertex labels by relation (3.1). Only the root labels are shown in the figure (To arrive at the form of the root label of $T_1 \circ T_2$ given here, the right-distributivity, cf. property (S6), is used)

The general idea developed in this section is as follows: The edges of the rooted trees are labeled by functions in a subset of the full transformation semigroup ${\mathcal {T}}({\mathfrak {X}})$. Each vertex is labelled by the sum of the functions on the outgoing edges multiplied with the functions of the respective head vertices (Definition 3.1, see Fig. 4 for an illustration). Moreover, there are operations of addition and multiplication (Definition 3.3 and Fig. 5) on the set of decorated rooted trees that are compatible with the addition and multiplication of the semigroup elements on the root (Lemma 3.6). The addition of two trees is performed by identifying their roots, and the multiplication is given by replacing the leaves of first tree with copies the second tree. Finally, to establish a relation to the semigroup models ${\mathcal {S}}^R$ and ${\mathcal {S}}$, the edge labels are chosen from the generating sets $\{\phi _r\}_{r \in R} \cup \{0\}$ and $\{\phi _x\}_{x \in X} \cup \{0\}$, respectively. This idea is also sketched in the mathematical outline in the introductory Sect. 1. The main Theorem 3.8 of this section establishes that both classes of decorated rooted trees are compatible with the algebraic structure of the semigroup models. The merit of this construction is that the F and RAF properties of a CRS can be reformulated in terms of decorated rooted trees and then directly cast into the language of semigroup models (Lemma 3.13).

The following notations and conventions with regard to rooted trees will be used. Let $T=(V,E,t)$ be a rooted tree with vertex set *V*, edge set $E \subset V \times V$ and root $t \in V$. Edges $(v,w) \in E$ are directed from *v* to *w*. Here, *v* is called the *tail* of *e* and *w* is its *head*. For each vertex $v \in V$, let $\text {ch}(v) \subset V$ denote the set of *children* of *V*, which is defined as $\text {ch}(v) := \{w \in V \text { such that }(v,w) \in E \}$. Also, denote by $T_v$ the subtree of *T* rooted at the vertex *v*. The *level* $\text {lv}(v)$ of a vertex *v* is the length of the path from the root to *v* and $\text {lv}_n(T) \subset V$ denotes the set of all vertices of a given level *n*. The notation $\textrm{ht}(T)$ denotes the *height* of the tree, i.e. the length of the longest path from the root to a leaf. Finally, $\textrm{lf}(T)$ is the set of all leaves of *T*, which is given by $\textrm{lf}(T) := \{v \in V \text { such that }\text {ch}(v) = \emptyset \}$. An edge $(v,w) \in E$ is said to be *terminal* if the vertex *w* is a leaf.

### Definition 3.1

For any subset $A \subset {\mathcal {T}}({\mathfrak {X}})$ of the full transformation semigroup ${\mathcal {T}}({\mathfrak {X}})$, an *A-decorated rooted tree* $T = (A,V,E,t,\omega _V,\omega _E)$ is a finite rooted tree with vertex set *V*, edge set *E*, a root $t \in V$ and two maps

$$\begin{aligned} \omega _V&: V \longrightarrow {\mathcal {T}}({\mathfrak {X}}) \\ \omega _E&: E \longrightarrow A, \end{aligned}$$

where $\omega _V$ is recursively given by

3.1 $$\begin{aligned} \omega _V(v) = {\left\{ \begin{array}{ll} \text {id}\mid _{{\mathfrak {X}}} &{}\text {if }v \in \text {lf}(T) \\ \sum _{w \in \text {ch}(v)} \omega _E((v,w)) \circ \omega _V(w) \qquad &{}\text {else}. \end{array}\right. } \end{aligned}$$

The addition and multiplication in the definition of $\omega _V$ takes place inside ${\mathcal {T}}({\mathfrak {X}})$ as previously defined (cf. Equations (2.2) and (2.3)). Figure 4 illustrates this construction.

Decorated rooted trees will be referred to as trees. For the set of edge labels $A \subset {\mathcal {T}}({\mathfrak {X}})$, denote the set of all *A*-decorated trees by ${\mathfrak {T}}(A)$. Also denote the set of all *A*-decorated trees of height *n* by ${\mathfrak {T}}(A)^n$ and of height at most *n* by ${\mathfrak {T}}(A)^{\le n}$. A subtree is defined as follows.

### Definition 3.2

A decorated rooted tree $T' = (A,V',E',t',\omega '_{V'},\omega '_{E'}) \in {\mathfrak {T}}(A)$ is a *subtree* of $T = (A,V,E,t,\omega _V,\omega _E) \in {\mathfrak {T}}(A)$ if there exists an injective map of rooted trees

$$\begin{aligned} g: (V',E',t') \rightarrow (V,E,t), \end{aligned}$$

which respects the labels on the edges, i.e.

$$\begin{aligned} \omega '_{E'}(e') = \omega _E(g(e')) \end{aligned}$$

holds for all $e' \in E'$.

The set ${\mathfrak {T}}(A)$ is equipped with two operations: Loosely speaking, given two trees $T_1, T_2 \in {\mathfrak {T}}(A)$, their sum is defined by identifying the roots of $T_1$ and $T_2$ and their product by replacing each leaf of $T_1$ with a copy of $T_2$.

### Definition 3.3

Let $T_1,T_2 \in {\mathfrak {T}}(A)$ be two *A*-decorated rooted trees given by the data $T_1 = (A,V_1,E_1,t_1,\omega _{V1},\omega _{E1})$ and $T_2 = (A,V_2,E_2,t_2,\omega _{V2},\omega _{E2})$. Define the tree $T^+ := T_1 + T_2$ with data $T^+ = (A,V^+,E^+,t^+,\omega _V^+,\omega _E^+)$ by identifying the roots of the two trees, i.e. byThere is a canonical map

$$\begin{aligned} \epsilon ^+: (V_1 \times V_1) \sqcup (V_2 \times V_2) \hookrightarrow (V_1 \sqcup V_2) \times (V_1 \sqcup V_2) \twoheadrightarrow V^+ \times V^+. \end{aligned}$$

The edge set $E^+$ is defined as

$$\begin{aligned} E^+ := \epsilon ^+(E_1 \sqcup E_2) \end{aligned}$$

with the decoration map

3.2 $$\begin{aligned} \omega _E^+(e) = {\left\{ \begin{array}{ll} \omega _{E1}((\epsilon ^+)^{-1}(e)) &{}\text {if }(\epsilon ^+)^{-1}(e) \in E_1\\ \omega _{E2}((\epsilon ^+)^{-1}(e)) &{}\text {if }(\epsilon ^+)^{-1}(e) \in E_2. \end{array}\right. } \end{aligned}$$

Because the restriction of $\epsilon ^+$ to $E_1 \sqcup E_2$ is one-to-one, this map is well-defined. The map $\omega _V^+$ is given by the relation (3.1) with $E^+$ and $\omega _E^+$ instead of *E* and $\omega _E$. The construction is illustrated in Fig. 5A.

Moreover, define the tree $T^{\circ } := T_1 \circ T_2$ with data $T^{\circ } = (A,V^{\circ },E^{\circ },t^{\circ },\omega _V^{\circ },\omega _E^{\circ })$ by replacing each leaf of $T_1$ with a copy of $T_2$. The data on $T^{\circ }$ is given as follows.where the equivalence relation $\sim$ relates each leaf $l \in \text {lf}(T_1) \subset V_1$ with the root $t_2 \in V_2$ of the respective copy of $V_2$ indexed by *l*. Again, there is a canonical map

$$\begin{aligned} \epsilon ^{\circ }: (V_1 \times V_1) \sqcup \coprod _{l \in \text {lf}(T_1)} (V_2 \times V_2) \rightarrow V^{\circ } \times V^{\circ } \end{aligned}$$

and the edge set is defined as $E^{\circ } := \epsilon ^{\circ }(E_1 \sqcup \coprod _{l \in \text {lf}(T_1)} E_2)$. The restriction of $\epsilon ^{\circ }$ to $E_1 \sqcup \coprod _{l \in \text {lf}(T_1)} E_2$ is one-to-one such that $\omega _E^{\circ }$ is defined analogously to $\omega _E^+$ as in (3.2). The map $\omega _V^{\circ }$ is defined by the relation (3.1) using $E^{\circ }$ and $\omega _E^{\circ }$ instead of *E* and $\omega _E$. This construction is illustrated in Fig. 5B.

The set ${\mathfrak {T}}(A)$, together with the two operations $\circ$ and $+$, is referred to as the *tree algebra* ${\mathfrak {T}}(A)$.

### Remark 3.4

It follows directly from the definition of the addition and multiplication of trees that the operations are associative. Moreover, the addition is commutative and the right distributivity

$$\begin{aligned} (T_1 + T_2) \circ T_3 = T_1 \circ T_3 + T_2 \circ T_3 \end{aligned}$$

holds by construction.

The algebraic structure on ${\mathfrak {T}}(A)$ thus defined is compatible with the algebraic structure on ${\mathcal {T}}({\mathfrak {X}})$ by mapping a tree $T \in {\mathfrak {T}}(A)$ to the label on its root via the following map.

### Definition 3.5

The map $ev: {\mathfrak {T}}(A) \longrightarrow {\mathcal {T}}({\mathfrak {X}})$ is called *evaluation map* and is defined as3.3

### Lemma 3.6

The map $ev: {\mathfrak {T}}(A) \longrightarrow {\mathcal {T}}({\mathfrak {X}})$ is a homomorphism with respect to addition $+$ and multiplication $\circ$.

### Proof

The notation from Definition 3.3 is used. Let $T_1,T_2 \in {\mathfrak {T}}(A)$ be two *A*-decorated rooted trees.

Let $T^+ = T_1 + T_2$. By construction of $T^+$, the projection $\pi : V_1 \sqcup V_2 \rightarrow V^+$ is injective on all vertices except on the root. Moreover, $\pi$ respects the level of a vertex, i.e. $\text {lv}(v) = \text {lv}(\pi (v))$, and the decoration function for vertices *v* of level 1 satisfies

$$\begin{aligned} \omega _V^+(v) = {\left\{ \begin{array}{ll} \omega _{V1}(\pi ^{-1}(v)) &{}\text {if }\pi ^{-1}(v) \in V_1\\ \omega _{V2}(\pi ^{-1}(v)) &{}\text {if }\pi ^{-1}(v) \in V_2. \end{array}\right. } \end{aligned}$$

This yields the homomorphism property for addition

$$\begin{aligned} \omega _V^+(t^+)&= \sum _{v \in \text {lv}_1(T^+)} \omega _E^+ ((t^+,v)) \circ \omega _V^+(v) \\&= \sum _{\begin{array}{c} v \in \text {lv}_1(T^+), \\ \pi ^{-1}(v) \in V_1 \end{array}} \omega _{E1}((t_1,v)) \circ \omega _{V1}(\pi ^{-1}(v)) \\&\quad +\sum _{\begin{array}{c} v \in \text {lv}_1(T^+), \\ \pi ^{-1}(v) \in V_2 \end{array}}\omega _{E2}((t_2,v)) \circ \omega _{V2}(\pi ^{-1}(v)) = \omega _{V1}(t_1) + \omega _{V2}(t_2). \end{aligned}$$

Let $T^{\circ } = T_1 \circ T_2$. By construction, $T_1$ is a subtree of $T^{\circ }$ and thus the respective vertices and edges of $T^{\circ }$ and $T_1$ can be identified. It is now shown inductively that for all $v \in T_1$, considered as a subtree of $T^{\circ }$, the relation

3.4 $$\begin{aligned} \omega _V^{\circ }(v) = \omega _{V1}(v) \circ \omega _{V2}(t_2) \end{aligned}$$

holds. For all leaves $l \in \text {lv}(T_1)$, the relation

$$\begin{aligned} \omega _V^{\circ }(l) = \omega _{V2}(t_2) = \text {id}\mid _{{\mathfrak {X}}} \circ \omega _{V2}(t_2) = \omega _{V1}(l) \circ \omega _{V2}(t_2) \end{aligned}$$

holds by construction. For the induction from vertex level *n* (with $1 \le n \le \text {ht}(T_1)$ to $n-1$, let $v \in V_1 \setminus \text {lf}(T_1)$ be a vertex of level $n-1$. The recursion (3.1) yields

$$\begin{aligned} \omega ^{\circ }_V(v)&= \sum _{w \in \text {ch}(v) \cap V_1} \omega ^{\circ }_E((v,w)) \circ \omega ^{\circ }_V(w) \\&= \sum _{w \in \text {ch}(v) \cap V_1} \omega _{E1}((v,w)) \circ \omega _{V1}(w) \circ \omega _{V2}(t_2) \\&= \left[ \sum _{w \in \text {ch}(v) \cap V_1} \omega _{E1}((v,w)) \circ \omega _{V1}(w) \right] \circ \omega _{V2}(t_2) \\&= \omega _{V1}(v) \circ \omega _{V2}(t_2), \end{aligned}$$

where $\omega ^{\circ }_E((v,w)) = \omega _{E1}((v,w))$ holds by definition, the second line is the induction hypothesis, and the third line follows from the right distributivity of the operations, cf. property (S6). In particular, the homomorphism property $\omega _V^{\circ }(t^+) = \omega _{V1}(t_1) \circ \omega _{V2}(t_2)$ holds. $\square$

Of particular importance are the trees decorated by the generating sets $\{ \phi _x \}_{x \in X} \cup \{0\}$ and $\{ \phi _r \}_{r \in R} \cup \{0\}$ of ${\mathcal {S}}^R$ and ${\mathcal {S}}$. The respective tree algebras are denoted by

$$\begin{aligned} {\mathfrak {T}}&:= {\mathfrak {T}}(\{ \phi _x \}_{x \in X} \cup \{0\}), \\ {\mathfrak {T}}^R&:= {\mathfrak {T}}(\{ \phi _r \}_{r \in R} \cup \{0\}). \end{aligned}$$

There is a map with nice algebraic properties between the tree algebraswhich is defined based on the relation $\phi _x = \sum _{(x,r) \in C} \phi _r$ between the edge labels. First, $\tau$ maps the trivial tree with one vertex in ${\mathfrak {T}}$ to the trivial tree in ${\mathfrak {T}}^R$. Next, let $T_{\phi }$ be the decorated rooted tree with one edge which is labelled by $\phi$. The tree $T_{\phi }$ is said to be the *atomic tree with label* $\phi$. For an atomic tree $T_{\phi _x} \in {\mathfrak {T}}$, the label function $\phi _x$ can be uniquely decomposed as a sum of functions corresponding to reactions according to its definition, cf. Equation (2.4):

$$\begin{aligned} \phi _x = \sum _{(x,r) \in C} \phi _r. \end{aligned}$$

Thus, $\tau (T_{\phi _x})$ is defined as the sum of the corresponding atomic trees

$$\begin{aligned} \tau (T_{\phi _x}) := \sum _{(x,r) \in C} T_{\phi _r}. \end{aligned}$$

A tree $T \in {\mathfrak {T}}$ of height one can be written as a finite sum of atomic trees, i.e. $T = \sum _{j=1}^m T_{\phi _{x_j}}$, and the map $\tau$ on ${\mathfrak {T}}^1$ is defined as

$$\begin{aligned} \tau (T) := \sum _{j=1}^m \tau ( T_{\phi _{x_j}}). \end{aligned}$$

An arbitrary tree $T \in {\mathfrak {T}}^n$ of height *n* can be written as $T = \sum _{j=1}^m T_{\phi _{x_j}} \circ T_j$ for atomic trees $T_{\phi _{x_j}}$ and trees $T_j \in {\mathfrak {T}}^{\le (n-1)}$ of height $\le (n-1)$. The map $\tau$ is defined recursively as

3.5 $$\begin{aligned} \tau (T) := \sum _{j=1}^m \tau ( T_{\phi _{x_j}}) \circ \tau (T_j). \end{aligned}$$

**Fig. 6:**
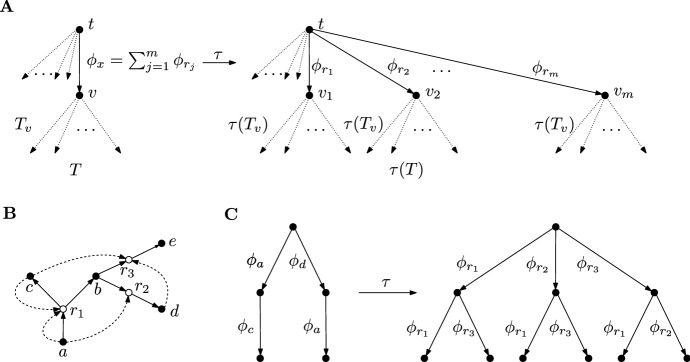
Illustration of the construction of the map $\tau : {\mathfrak {T}} \rightarrow {\mathfrak {T}}^R$. **A** Illustration of the general procedure of replacing an edge of $T \in {\mathfrak {T}}$ with label $\phi _x$ by an edge for each summand in $\phi _x = \sum _{(x,r) \in C} \phi _r = \sum _{j=1}^m \phi _{r_j}$ with labels $\phi _{r_j}$. This is performed recursively starting with the terminal edges and working upwards toward the root. **B** An example CRS with the functions of chemicals given by $\phi _a = \phi _{r_1} + \phi _{r_2}$, $\phi _c = \phi _{r_1} + \phi _{r_3}$ and $\phi _d = \phi _{r_3}$. **C** The map $\tau$ applied to the tree *T* on the left for the CRS featured in B

The substitution process is illustrated in Fig. 6A and an example of the construction $T \mapsto \tau (T)$ for the CRN in Fig. 6B is given in Fig. 6C.

### Lemma 3.7

The map $\tau : {\mathfrak {T}} \rightarrow {\mathfrak {T}}^R$ defined above is a homomorphism with respect to the addition and multiplication of trees. Moreover, the label of the root $\omega _V(t)$ is invariant under $\tau$ for any tree $T \in {\mathfrak {T}}$, i.e., using the evaluation map defined in (3.3), the relation

$$\begin{aligned} ev(T) = ev(\tau (T)) \end{aligned}$$

holds.

### Proof

Let $T_1, T_2$ be two nontrivial trees in ${\mathfrak {T}}$. They can be written as $T_1 = \sum _{j=1}^m T_{\phi _{x_j}} \circ T_j$ and $T_2 = \sum _{j=m+1}^l T_{\phi _{x_j}} \circ T_j$ with atomic trees $T_{\phi _{x_j}}$. Their sum is given by $T_1 + T_2 = \sum _{j=1}^l T_{\phi _{x_j}} \circ T_j$ and the compatibility of $\tau$ with respect to addition follows from the definition (3.5) and from the associativity of addition $\tau (T_1 + T_2) = \sum _{j=1}^l \tau (T_{\phi _{x_j}}) \circ \tau (T_j) = \tau (T_1) + \tau (T_2)$.

The compatibility of $\tau$ with respect to multiplication is shown inductively on $\text {ht}(T_1)$. If $\text {ht}(T_1) = 1$, then $T_1 = \sum _{j=1}^m T_{\phi _{x_j}}$ and $T_1 \circ T_2 = ( \sum _{j=1}^m T_{\phi _{x_j}} ) \circ T_2 = \sum _{j=1}^m ( T_{\phi _{x_j}} \circ T_2 )$ by Remark 3.4. Thus, the property $\tau (T_1 \circ T_2) = \sum _{j=1}^m ( \tau (T_{\phi _{x_j}}) \circ \tau (T_2) ) = ( \sum _{j=1}^m \tau (T_{\phi _{x_j}})) \circ \tau (T_2) = \tau (T_1) \circ \tau (T_2)$ follows from the definition (3.5) and the right distributivity of the tree algebra ${\mathfrak {T}}^R$. Let $\text {ht}(T_1) = n$ and let $T_1$ be given by expression $T_1 = \sum _{j=1}^m T_{\phi _{x_j}} \circ T_j$ as above with $\text {ht}(T_j) \le (n-1)$. Then $T_1 \circ T_2 = ( \sum _{j=1}^m T_{\phi _{x_j}} \circ T_j ) \circ T_2 = \sum _{j=1}^m ( T_{\phi _{x_j}} \circ T_j \circ T_2 )$ yields

$$\begin{aligned} \tau (T_1 \circ T_2)&= \sum _{j=1}^m \left[ \tau (T_{\phi _{x_j}}) \circ \tau (T_j \circ T_2) \right] \\&= \sum _{j=1}^m \left[ \tau (T_{\phi _{x_j}}) \circ \tau (T_j) \circ \tau (T_2) \right] \\&= \sum _{j=1}^m \left[ \tau (T_{\phi _{x_j}}) \circ \tau (T_j) \right] \circ \tau (T_2) = \tau (T_1) \circ \tau (T_2), \end{aligned}$$

where the first line follows from the definition (3.5), the second line from the induction hypothesis, and the third line from the right distributivity of the tree algebra.

The invariance of the root label holds for an atomic tree as $ev(T_{\phi _x}) = \phi _x$ and $ev(\tau (T_{\phi _x})) = ev( \sum _{(x,r) \in C} T_{\phi _r}) = \sum _{(x,r) \in C} \phi _r = \phi _x$. It extends to the trees of height 1 by the associativity of the addition of trees and elements in ${\mathcal {T}}({\mathfrak {X}})$. For a tree of arbitrary height this is verified inductively. Let $T = \sum _{j=1}^m T_{\phi _{x_j}} \circ T_j$. Its root label is determined by (3.1) as $ev(T) = \sum _{j=1}^m \phi _{x_j} \circ ev(T_j)$. The root label of $\tau (T)$ is given by

$$\begin{aligned} ev(\tau (T)) = ev\left( \sum _{j=1}^m \tau (T_{\phi _{x_j}}) \circ \tau (T_j)\right) = \sum _{j=1}^m \phi _{x_j} \circ ev(\tau (T_j)), \end{aligned}$$

which agrees with *ev*(*T*) by induction hypothesis. $\square$

These constructions yield the following central theorem.

### Theorem 3.8

With the maps *ev* and $\tau$ defined above and inclusion ${\iota :\mathcal {S} \cup \{ \text {id}\mid _{\mathfrak {X}}\}} \rightarrow \mathcal {S}^R \cup \{ \text {id}\mid _{\mathfrak {X}} \}$ , the following diagram commutes 
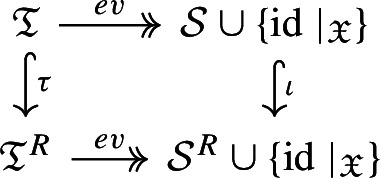


and all maps are homomorphisms. Moreover, the evaluation maps *ev* are surjective.

### Proof

The homomorphism property follows from the Lemmata 3.6 and 3.7. The commutativity of the diagram has also been proven in Lemma 3.7. The evaluation maps are surjective because the generators $\{\phi _x\}_{x \in X} \cup \{0\}\subset {\mathcal {S}}$ and ${\{\phi _r\}_{x \in R} \cup \{0\} \subset {\mathcal {S}}^R}$ have preimages given by the atomic trees $\{T_{\phi _x}\}_{x \in X} \cup \{T_0\} \subset {\mathfrak {T}}$ and ${\{T_{\phi _r}\}_{r \in R} \cup \{T_0\} \subset {\mathfrak {T}}^R}$ combined with the fact that the tree algebras are closed under the operations of addition and multiplication. $\square$

The finiteness of ${\mathcal {S}}$ yields the following corollary.

### Corollary 3.9

There is an *N* such that the set of trees of height at most ${\mathfrak {T}}^{\le N}$ maps surjectively onto ${\mathcal {S}} \cup \{ \text {id}\mid _{{\mathfrak {X}}} \}$.

Moreover, Theorem 3.8 implies that the elements of ${\mathcal {S}}$ and ${\mathcal {S}}^R$ can be represented as decorated rooted trees by lifting the respective semigroup elements via the homomorphism *ev*.

### Definition 3.10

A *tree representative* of an element $\phi \in {\mathcal {S}}$ is an element $T \in {\mathfrak {T}}$ such that $ev(T) = \phi$. The representative is called *minimal* if it has no subtree $T'$ such that $ev(T') = \phi$. The analogous definition holds for tree representatives of elements of ${\mathcal {S}}^R$ in ${\mathfrak {T}}^R$.

### Remark 3.11

(Biochemical interpretation of a tree) . A tree $T \in {\mathfrak {T}}^R$ of level *n* corresponds to a “reaction mechanism” of the network which can be described as follows: The reactions at the terminal edges are carried out and their products are supplied to their tail vertices. For each vertex, once it has received the products from all its outgoing edges, these products act as reactants for the reaction on its incoming edge. This procedure is carried out iteratively for the levels of the tree and therefore takes *n* steps for a tree of height *n*. For a tree $T \in {\mathfrak {T}}$, the respective reaction mechanism is the reaction mechanism described $\tau (T) \in {\mathfrak {T}}^R$.

Finally, the decorated rooted trees in ${\mathfrak {T}}^R$ can be used to reformulate the F property given in Definition 2.4. In particular, the condition (F) can be encoded in a tree:

### Definition 3.12

Let $x \in X_F$ be a chemical for which the condition (F) holds. Let $R_1,\dotsc ,R_n$ be the sets of reactions featured in (F) and denote by $T_{\phi _r}$ the atomic trees for the functions $\phi _r$. Define the trees $T_1^R,\dotsc ,T_n^R \in {\mathfrak {T}}^R$ inductively as follows: Let

3.6 $$\begin{aligned} T_1^R := \sum _{r \in R_1} T_{\phi _r}, \end{aligned}$$

and for $$1 \le i < n$$

3.7 $$\begin{aligned} T_{i+1}^R := \sum _{r \in \bigcup _{j=1}^{i+1} R_j} T_{\phi _r} \circ T_i^R. \end{aligned}$$

The tree $T_n^R$ is said to be the *F-tree* for the element *x*. It is denoted by $T^R(x)$.

### Lemma 3.13

Let $x \in X_F$ be a chemical for which the condition (F) holds. Then for the F-tree $T^R(x)$, the relation

$$\begin{aligned} x \in ev\left( T^R(x)\right) (\emptyset ) \end{aligned}$$

holds. In other words, $T^R(x)$ represents a reaction mechanism that produces *x* from the food set.

### Proof

Let $T_i^R$ be the trees from Definition 3.12 with $T_n^R =T^R(x)$. It will be shown inductively that

3.8 $$\begin{aligned} \bigcup _{j=1}^{i} \text {ran}(R_j) \subset ev\left( T_i^R\right) (\emptyset ) \cup F \end{aligned}$$

holds for all $i=1,\dotsc ,n$ and therefore

3.9 $$\begin{aligned} \bigcup _{j=1}^{i+1} \text {dom}(R_j) \subset ev\left( T_i^R\right) (\emptyset ) \cup F \end{aligned}$$

holds for all $i=1,\dotsc ,n-1$. The inclusion (3.9) follows from (3.8) together with the conditions (F1) and (F2). Then, the claim $x \in ev\left( T_n^R\right) (\emptyset )$ will follow from the inclusion (3.8) together with the condition (F3).

For $i=1$, the definition of $T_1^R$ gives

$$\begin{aligned} ev\left( T_1^R\right) (\emptyset ) \cup F = \sum _{r \in R_1} \phi _r (\emptyset ) \cup F. \end{aligned}$$

From condition (F1), i.e. $\text {dom}(R_1) \subset F$, it follows that $\text {ran}(R_1) = \sum _{r \in R_1} \phi _r(\emptyset ) \subset ev\left( T_1^R\right) (\emptyset ) \cup F$. And from condition (F2), i.e. $\text {dom}(R_2) \subset \text {ran}(R_1) \cup F$, together with (F1), it follows that $\text {dom}(R_1) \cup \text {dom}(R_2) \subset ev\left( T_1^R\right) (\emptyset ) \cup F$.

For $i+1$, one obtains

$$\begin{aligned} ev\left( T_{i+1}^R\right) (\emptyset ) \cup F&= \sum _{r \in \bigcup _{j=1}^{i+1} R_j} \phi _r\left( ev\left( T_i^R\right) (\emptyset )\right) \cup F \\&\supset \bigcup _{j=1}^{i+1} \text {ran}(R_j), \end{aligned}$$

where the final inclusion is obtained from the induction hypothesis $\bigcup _{j=1}^{i+1} \text {dom}(R_j) \subset ev\left( T_i^R\right) (\emptyset ) \cup F$. The conditions (F1) and (F2) imply now that $\bigcup _{j=1}^{i+2} \text {dom}(R_j) \subset ev\left( T^R_{i+1}\right) (\emptyset ) \cup F$ for $i \le n-2$. $\square$

## Characterization of self-sustaining and self-generating CRS

In this section, the representation of semigroup elements as trees is used to derive a succinct expression for the maximal RAF set of chemicals of a CRS as the fixed point of the generative dynamics $Y \mapsto \Phi _Y(\emptyset )$ with the initial condition $Y_0 = X_F$ (Theorem 4.9). In Sect. 4.1, it is shown that a CRS if RAF if and only if $\Phi _{X_F}(\emptyset ) = X_F$ holds (Theorem 4.1) and that the condition $\Phi _{X_F'}(\emptyset ) = X_F'$ is sufficient for a set of chemicals $X_F' \subset X_F$ to be a RAF set of chemicals (Proposition 4.3). The latter statement is the key statement to prove that the fixed point of the dynamics, which is introduced in Sect. 4.2, satisfies the desired properties. The importance of fixed points of the dynamics as functionally closed and therefore biologically relevant entities is discussed in Sect. 4.3.

This whole section follows a logical structure which is analogous the structure of Section 4 in Loutchko (2022), where the analogous statements are proven for self-sustaining CRS. However, the treatment of self-generating CRS is technically more involved, which is forced by the fact that the F property is more involved than the self-sustainment property of a CRS, cf. Remark 2.6.

Throughout this section, fix a CRS (*X*, *R*, *C*, *F*) and let ${\mathcal {S}}$ be its semigroup model.

### Characterization of CRS with the RAF property

A CRS with the RAF property can be conveniently characterized via the set of chemicals generated by the maximal function of its semigroup model from the food set.

#### Theorem 4.1

A CRS is RAF if and only if the maximal function $\Phi _{X_F}$ of its semigroup model satisfies

4.1 $$\begin{aligned} \Phi _{X_F}(\emptyset ) = X_F. \end{aligned}$$

#### Proof

If the CRS is RAF, then by Lemma 3.13, the function $ev(T^R(x))$ satisfies $x \in ev(T^R(x))(\emptyset )$ for all $x \in X_F$. This function is an element of ${\mathcal {S}}^R$ but not of ${\mathcal {S}}$ in general. The RAF property allows to construct a tree $T(x) \in {\mathfrak {T}}$ such that $ev(T^R(x)) \le ev(T(x))$ and thus $x \in ev(T(x))(\emptyset )$: Choose a catalyst $y(r) \in X$ for each reaction $r \in R_i$ for all $R_i$ featured in the condition (F) for $x \in X_F$. In analogy to the formulae (3.6) and (3.7), define

4.2 $$\begin{aligned} T_1 := \sum _{r \in R_1} T_{\phi _{y(r)}}, \end{aligned}$$

and for $$1<i<n$$:

4.3 $$\begin{aligned} T_{i+1} := \sum _{r \in \bigcup _{j=1}^{i+1} R_j} T_{\phi _{y(r)}} \circ T_i \end{aligned}$$

with the atomic trees $T_{\phi _{y(r)}} \in {\mathfrak {T}}$ and set $T(x) := T_n$. The properties (S1), (S2) and (S3) ensure that $ev(T^R(x)) \le ev(T(x))$. The function

$$\begin{aligned} \Phi := \sum _{x \in X_F} ev(T(x)) \end{aligned}$$

satisfies $X_F \subset \Phi (\emptyset )$ and thus the equality $\Phi (\emptyset ) = X_F$ holds. Therefore, $\Phi$ is the maximal function $\Phi _{X_F}$ of ${\mathcal {S}}$ and the claim $\Phi _{X_F}(\emptyset ) = X_F$ holds.

To prove the reverse, assume that $\Phi _{X_F}(\emptyset ) = X_F$ holds. Choose a representative $T \in {\mathfrak {T}}$ for $\Phi _{X_F}$, i.e. a tree *T* such that $ev(T)= \Phi _{X_F}$, and consider its image $\tau (T) \in {\mathfrak {T}}^R$. Fix a chemical $x \in X_F$. By Theorem 3.8, the relation

$$\begin{aligned} x \in ev(\tau (T))(\emptyset ) \end{aligned}$$

holds. Choose a subtree $T^{min}(x) \in {\mathfrak {T}}^R$ of $\tau (T)$ which is minimal under the condition

4.4 $$\begin{aligned} x \in ev(T^{min}(x))(\emptyset ). \end{aligned}$$

The existence of $T^{min}(x)$ follows from the existence of $\tau (T)$. The sets $R_1,\dotsc ,R_n$ featured in the condition (F) are constructed as follows: Let the height of $T^{min}(x)$ be *n* and define the set $R_i$ to contain the reaction corresponding to the labels of all edges whose heads have level $n+1-i$ for $1 \le i \le n$, i.e.

4.5 $$\begin{aligned} R_i := \{r \in R \text { such that } \phi _r = \omega _E(e) \text { for some } e \in \text {elv}_{n+1 -i}(T^{min}(x)) \}, \end{aligned}$$

where $\omega _E$ is the decoration function for the edges of $T^{min}(x)$ and $\text {elv}_m(T^{min}(x))$ denotes the set of edges of level *m*, which are all the edges whose head vertex has level *m*. By the minimality of $T^{min}(x)$, the conditions (F1) and (F2) must be satisfied (reactions in any of the $R_i$ which do not satisfy the conditions could be omitted from the tree without violating the condition (4.4) thus contradicting the minimality of $T^{min}(x)$). The condition (F3) holds by construction of $T^{min}(x)$. Finally, all reactions appearing as edge labels of $T^{min}(x)$, and thereby all reactions in the sets $R_1,\dotsc ,R_n$, are catalyzed by construction of $\tau (T)$ This concludes the proof. $\square$

#### Corollary 4.2

If $X_F' \subset X_F$ is a RAF set of chemicals, then the inclusion

$$\begin{aligned} X_F' \subset \Phi _{X_F'}(\emptyset ) \end{aligned}$$

holds.

#### Proof

The maximal function $\Phi '_{X'_F}$ of the semigroup model ${\mathcal {S}}'(X',R\mid _{X'})$ satisfies $\Phi '_{X'_F}(\emptyset ) = X'_F$ by Theorem 4.1. Its extension $ext(\Phi '_{X'_F})$, defined in Remark 2.14, satisfies $ext(\Phi '_{X'_F})(\emptyset ) = X'_F$ by definition. The relation (2.9) gives

$$\begin{aligned} ext(\Phi '_{X'_F}) \le \Phi _{X'_F} \end{aligned}$$

and yields the claim when the functions above are applied to the empty set. $\square$

For a RAF set of chemicals $X'_F$, the inclusion $X'_F \subset \Phi _{X'_F}(\emptyset )$ can be strict. An example would be the set $X_F' = \{c,d\}$ in Fig. 2, where one verifies that $\Phi _{\{c,d\}}(\emptyset ) = \{c,d,e\}$. Therefore the equality $X'_F = \Phi _{X'_F}(\emptyset )$ is not always satisfied for a RAF set of chemicals. However, it provides a sufficient condition.

#### Proposition 4.3

If the equality $X'_F = \Phi _{X'_F}(\emptyset )$ holds for a set of chemicals $X'_F \subset X_F$, then $X'_F$ is a RAF set of chemicals.

#### Proof

The proof is analogous to the second part of the proof of Theorem 4.1. As in that proof, let $T \in {\mathfrak {T}}$ be a tree representative for the function $\Phi _{X'_F} \in {\mathcal {S}}$ of minimal height and let $T^{min}(x) \in {\mathfrak {T}}^R$ be a minimal subtree of $\tau (T)$ that satisfies $x \in ev(T^{min}(x))(\emptyset )$ for $x \in X'_F$ and has the same height as *T*. Moreover, let *T* be chosen such that all its edge labels are contained in the generating set $\{\phi _x\}_{x \in X'}$ of ${\mathcal {S}}(X'_F)$ (this is always possible since *T* represents an element of ${\mathcal {S}}(X'_F)$). This leads to the sets of reactions $R_1,\dots ,R_n$ defined by (4.5) and satisfying the condition (F) (the verification of this condition is analogous to the verification in the proof of Theorem 4.1). One only needs to ensure that all reactions *r* contained in the $R_i$ satisfy $\text {supp}(r) \subset X'$, i.e. that they are elements of $R\mid _{X'}$, which is now shown:

The domain of each $R_i$ for $1 \le i \le n$ satisfies

$$\begin{aligned} \text {dom}(R_i) \subset \bigcup _{v \in \text {lv}_{n+1-i}(T^{min}(x))} \omega _V(v)(\emptyset ) \cup F, \end{aligned}$$

because the edges corresponding to the reactions with domains which are not contained in the set on the right hand side could be removed from $T^{min}(x)$, which would contradict its minimality (in the above formula, $\omega _V$ is the vertex decoration function of $T^{min}(x)$). Therefore, it follows inductively that

$$\begin{aligned} \text {ran}(R_i) \subset \bigcup _{v \in \text {lv}_{n-i}(T^{min}(x))} \omega _V(v)(\emptyset ) \cup F. \end{aligned}$$

Consider the functions

$$\begin{aligned} \phi _i^R:=\sum _{v \in \text {lv}_{n-i}(T^{min}(x))}\omega _V(v) \in {\mathcal {S}}^R, \end{aligned}$$

which satisfy $\text {ran}(R_i) \subset \phi _i^R(\emptyset ) \cup F$. By construction of $T^{min}(x)$ as a subtree of $\tau (T)$ of the same height, the function $\phi _i^R$ is bounded from above by corresponding function $\phi _i$ constructed from *T*

$$\begin{aligned} \phi _i:=\sum _{v \in \text {lv}_{n-i}(T)}\omega _V(v) \in {\mathcal {S}}. \end{aligned}$$

The $\phi _i$ are elements of ${\mathcal {S}}(X'_F)$ and are thus bounded from above by $\Phi _{X'_F}$. This leads to the inclusion

$$\begin{aligned} \text {ran}(R_i) \subset \phi _i^R(\emptyset ) \cup F \subset \phi _i(\emptyset ) \cup F \subset \Phi _{X'_F}(\emptyset ) \cup F = X'. \end{aligned}$$

Together with the properites (F1) and (F2), this yields $\text {supp}(R_i) \subset X'$ for all sets $R_i$. $\square$

This proposition will be used to show that the fixed points of the dynamics, defined in the next section, are RAF sets of chemicals.

### Generative dynamics on a semigroup model and identification of the maximal RAF set of chemicals

The generative discrete dynamics of a CRS is introduced and used to determine the maximal RAF set of chemicals. Starting with any set of chemicals $Y_0 \subset X_F$, there is a maximal function $\Phi _{Y_0}$ (Definition 2.12) that is supported on this set. By acting on the empty set, $\Phi _{Y_0}(\emptyset )$ gives all non-food chemicals that can be generated from the food set by using functionality supported only on $Y_0$ and the food set. The argument can be applied iteratively and gives rise to the following definition.

#### Definition 4.4

The *generative dynamics* of a CRS with the initial condition $Y_0 \subset X_F$ is generated by the propagator

4.6 $$\begin{aligned} {\mathcal {D}}^g : {\mathfrak {X}}&\rightarrow {\mathfrak {X}}\nonumber \\ Y&\mapsto \Phi _Y(\emptyset ), \end{aligned}$$

where $\Phi _Y$ is the function of $Y \subset X_F$. The dynamics generated by ${\mathcal {D}}^g$ is parametrized by ${\mathbb {Z}}_{\ge 0}$ as

$$\begin{aligned} Y_{n+1} = {\mathcal {D}}^g(Y_n) \text { for all } n \in {\mathbb {Z}}_{\ge 0}. \end{aligned}$$

The generative dynamics has analogous properties to the sustaining dynamics and the reader is referred to Section 4.2. in Loutchko (2022) for a more detailed discussion. Here, only the properties needed for the proof of the main theorem are given.

#### Remark 4.5

Due to the finiteness of the state space ${\mathfrak {X}}$, the dynamics either leads to a fixed point or to periodic behavior.

#### Definition 4.6

If the generative condition with initial condition $Y_0$ leads to a fixed point, the dynamics is said to *stabilize* and the fixed point is denoted by $Y_0^{*g}$.

#### Proposition 4.7

Let the dynamics be given by $(Y_n)_{n \in {\mathbb {Z}}_{\ge 0}}$. If $Y_1 \subset Y_0$, then

$$\begin{aligned} Y_{n+1} \subset Y_n \end{aligned}$$

holds for all $n \in {\mathbb {Z}}_{\ge 0}$ and the dynamics stabilizes. The analogous statement holds for the case that $Y_1 \supset Y_0$.

#### Proof

The proof proceeds by induction. By hypothesis $Y_1 \subset Y_0$ is satisfied. Let $Y_n \subset Y_{n-1}$. This implies the ordering of the respective functions $\Phi _{Y_n} \le \Phi _{Y_{n-1}}$ by the property (S8). Together with the property (S1) this gives

$$\begin{aligned} Y_{n+1} = \Phi _{Y_n}(\emptyset ) \subset \Phi _{Y_{n-1}}(\emptyset ) = Y_n. \end{aligned}$$

The dynamics is thus given by the decreasing chain of subsets $Y_0 \supset Y_1 \supset ... \supset Y_n \supset Y_{n+1} \dots$ and, because $X_F$ is finite, the chain stabilizes. The case $Y_1 \supset Y_0$ is treated analogously. $\square$

#### Lemma 4.8

Let $X'_F \subset X_F$ be a RAF set of chemicals and let *Y* be a set that satisfies $X'_F \subset Y \subset X_F$. Then the inclusion

$$\begin{aligned} X'_F \subset \Phi _Y(\emptyset ) \end{aligned}$$

holds.

#### Proof

The chain of inclusions

$$\begin{aligned} X'_F \subset \Phi _{X'_F}(\emptyset ) \subset \Phi _Y(\emptyset ) \end{aligned}$$

follows from the Corollary 4.2 and the property (S8). $\square$

Now the main theorem is stated and proven:

#### Theorem 4.9

(On the maximal RAF set of chemicals) The maximal RAF set of chemicals of a CRS is the fixed point of the generative dynamics $(Y_n)_{n \in {\mathbb {Z}}_{\ge 0}}$ with the initial condition $Y_0 = X_F$, i.e. it is the set $X_F^{*g}$.

#### Proof

It follows from Proposition 4.7 that the dynamics has a fixed point $X_F^{*g}$. By Proposition 4.3 this fixed point is a RAF set of chemicals. It remains to show the maximality of $X_F^{*g}$: For any RAF set of chemicals $X_F' \subset X_F$, the repeated application of Lemma 4.8 implies that $X_F' \subset Y_n$ for all $n \in {\mathbb {Z}}_{\ge 0}$ and therefore $X_F' \subset X_F^{*g}$. $\square$

#### Corollary 4.10

A CRS with a nilpotent semigroup $({\mathcal {S}},\circ )$ has no nontrivial RAF sets of chemicals^3^.

#### Proof

Let $X_F' \subset X_F$ be a nontrivial RAF set of chemicals. Then $X_F' \subset \Phi _{X_F'}(\emptyset )$ holds by Corollary 4.2 and thus the condition (S1) implies that

$$\begin{aligned} X_F' \subset \Phi _{X_F'}^n(\emptyset ) \end{aligned}$$

for any power of $\Phi _{X_F'}$, i.e. $\Phi _{X_F'}^n$ is nonzero for any $n \in {\mathbb {N}}$. $\square$

Nilpotent semigroups comprise the largest class of semigroups as any magma^4^ with the product of any three elements equal to zero is automatically a semigroup, cf. Satoh et al. (1994); Almeida (1995). The above corollary weeds out all nilpotent semigroups as candidates for semigroup models of self-generating CRS and states that such models are located in a more interesting class of semigroups.

#### Remark 4.11

(Connection to the RAF algorithm). Hordijk and Steel (2004) have presented an algorithm to find the maximal RAF set of reactions. It consists of a dynamics on the power set of reactions ${\mathcal {P}}(R)$ generated by $R' \mapsto \delta (\gamma (R'))$ with the initial condition $R_0 = R$. The following two operations are performed iteratively:

1. For a set $R' \subset R$, remove all reactions from $R'$ that have no catalyst in $\text {supp}(R')$ until no further reductions can be made. This yields the set $\gamma (R')$.
2. For a set $R' \subset R$, until no further reductions can be made, remove all reactions *r* from $R'$ that satisfy ${\text {dom}(r) \not \subset \Phi _{R'}(\emptyset ) \cup F}$, where $\Phi _{R'}$ is the maximal function of the semigroup model ${\mathcal {S}}^R(R') := \langle \phi _r \rangle _{r \in R'}$. This yields the set $\delta (R')$.

Note that (R2) has been rephrased here to suit the language of semigroup models. This is similar in spirit to the algorithm given in Theorem 4.9 by the generative dynamics $Y \mapsto \Phi _Y(\emptyset )$, where the sets of chemicals *Y* should be thought of as the support of $R'$ featured in the RAF algorithm. By forming the function $\Phi _Y$, all reactions without a catalyst in $Y = \text {supp}(R')$ are excluded, which corresponds to (R1). The application of the function $\Phi _Y$ to the empty set corresponds to the exclusion of all reactions without support in $\Phi _Y(\emptyset )$, i.e. to the step (R2).

### Functionally closed RAF sets of chemicals

In addition to the knowledge of the maximal RAF set of chemicals, the hierarchy of RAF subsets of chemicals plays an important role in the understanding of a CRS. Of particular importance are the RAF sets of chemicals which satisfy the fixed point equation for the dynamics and are termed *functionally closed* RAF sets of chemicals in this section. This is closely related to the notion of functionally closed sets of self-sustaining chemicals, which is developed in Loutchko (2022), Section 4.4.

If, for a set of chemicals $X_F' \subset X_F$, the inclusion $X_F' \subset \Phi _{X_F'}(\emptyset )$ is strict, then the set is not stable in the sense that it will produce additional chemicals over time. First, the chemicals in $Y_1 = \Phi _{X_F'}(\emptyset )$ will be generated from the food set, followed by chemicals in $Y_2 = \Phi _{Y_1}(\emptyset )$, etc. By Proposition 4.7, this dynamics stabilizes at the fixed point $X_F'^{*g}$, which contains the initial set of chemicals $X_F'$. Moreover, being a fixed point of the dynamics, $X_F'^{*g}$ satisfies

$$\begin{aligned} \Phi _{X_F'^{*g}}(\emptyset )=X_F'^{*g} \end{aligned}$$

and is thus a RAF set of chemicals by Proposition 4.7. The set $X_F'^{*g}$ is not able to further catalyze the generation of chemicals outside of $X_F'^{*g}$ from the food set and is thus functionally closed. This motivates the following definitions.

#### Definition 4.12

A set of chemicals $X'_F \subset X_F$ which satisfies

$$\begin{aligned} X'_F \subset \Phi _{X'_F}(\emptyset ) \end{aligned}$$

is called *pre-functionally closed RAF*. A set of chemicals $X'_F \subset X_F$ is said to be *functionally closed RAF* if it satisfies

$$\begin{aligned} X'_F = \Phi _{X'_F}(\emptyset ). \end{aligned}$$

The sets of all (pre-)functionally closed RAF sets of chemicals are denoted by

$$\begin{aligned} \mathfrak {pF}&:= \{ X_F' \subset X_F \text { such that } X'_F \subset \Phi _{X'_F}(X'_F) \} \\ {\mathfrak {F}}&:= \{ X_F' \subset X_F \text { such that } X'_F = \Phi _{X'_F}(X'_F) \} \subset \mathfrak {pF}. \end{aligned}$$

#### Definition 4.13

The *functional closure* of a pre-functionally closed RAF set of chemicals $X_F' \subset X_F$ is the fixed point $X_F'^{*g}$ of the generative dynamics.

Alternatively, the functional closure of a pre-functionally closed RAF set of chemicals can be characterized as follows:

#### Lemma 4.14

The functional closure $X_F'^{*g}$ of a pre-functionally closed RAF set of chemicals $X_F'$ is the unique minimal functionally closed RAF set of chemicals which contains $X_F'$.

#### Proof

Let *Y* be a minimal functionally closed RAF set which contains $X_F'$ and let $(Y_n)_{n \in {\mathbb {Z}}_{\ge 0}}$ be the generative dynamics with the initial condition $Y_0 = X_F'$ and fixed point $X_F'^{*g}$. Then $Y_n \subset Y$ holds for all $n \in {\mathbb {Z}}_{\ge 0}$, which can be verified by induction: For $n=0$, the claim holds by assumption and the inductive step is verified by

$$\begin{aligned} Y_{n+1} = \Phi _{Y_n} (\emptyset ) \subset \Phi _Y (\emptyset ) = Y, \end{aligned}$$

which follows from the property (S8). This implies that $X_F'^{*g} \subset Y$ and by the minimality of *Y*, the equality $X_F'^{*g} = Y$ must hold. $\square$

#### Remark 4.15

**Fig. 7:**
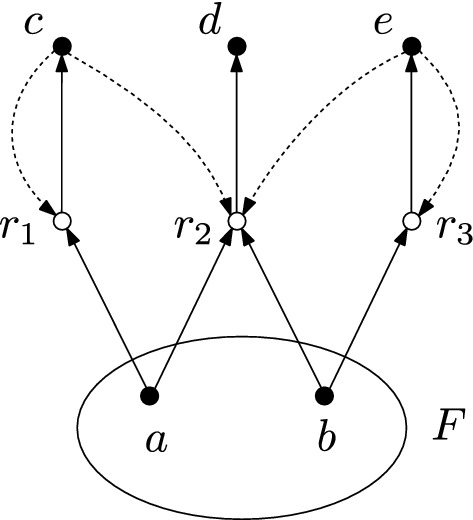
This CRS has the three functionally closed sets of chemicals given by $X_F = \{c,d,e\}$, $X_F' = \{c,d\}$ and $X_F'' = \{d,e,\}$. There is no unique minimal functionally closed set of chemicals which contains the set $\{d\}$

Note that the characterization of the functional closure of a pre-functionally closed RAF set of chemicals given by Lemma 4.14 does not extend to arbitrary sets, i.e. in general there does not exist a *unique* minimal functionally closed set of chemicals which contains *Y* for a arbitrary set of chemicals $Y \subset X_F^{*g}$. Figure 7 provides an illustration. The shown CRS is RAF and it has the functionally closed sets of chemicals given by $X_F = \{c,d,e\}$, $X_F' = \{c,d\}$ and $X_F'' = \{d,e\}$. For the set $\{d\}$, there exists no unique minimal functionally closed set of chemicals which contains it.

The concept of functional closure establishes a connection between the two sets $\mathfrak {pF}$ and ${\mathfrak {F}}$:

#### Lemma 4.16

The set ${\mathfrak {F}}$ is obtained by taking the functional closure of all elements of $\mathfrak {pF}$, i.e.

$$\begin{aligned} {\mathfrak {F}} = \{ Y^{*g} \text { for } Y \in \mathfrak {pF} \}. \end{aligned}$$

#### Proof

The $\subset$ inclusion holds because $\mathfrak {pF}$ contains all functionally closed RAF sets and this does not change when taking the functional closure. The $\supset$ inclusion holds because the set on the right hand side contains only functionally closed RAF sets by construction. $\square$

The connection to RAF sets of chemicals is given by the following theorem.

#### Theorem 4.17

For sets of chemicals, the functionally closed RAF property implies the RAF property, which in turn implies the pre-functionally closed RAF property, i.e., the following inclusions of sets hold: 
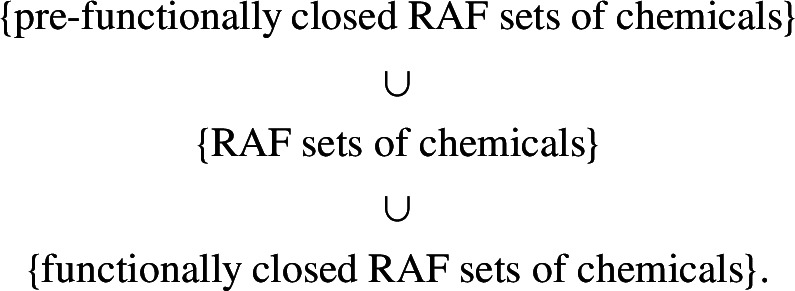


#### Proof

This follows directly from Proposition 4.3 and Corollary 4.2. $\square$

The set of all (pre-)functionally closed RAF sets of chemicals can be obtained by the following construction. Define the *reduced generative dynamics* by the propagator

$$\begin{aligned} {\mathcal {D}}^{rg} : {\mathfrak {X}}&\rightarrow {\mathfrak {X}}\nonumber \\ Y&\mapsto Y \cap \Phi _Y(\emptyset ). \end{aligned}$$

This dynamics always stabilizes, and the fixed point for the initial condition $Y_0$ is denoted as $Y_0^{*rg}$. The fixed point equation of this dynamics reads ${Y = Y \cap \Phi _Y(\emptyset )}$, which is equivalent to $Y \subset \Phi _Y(\emptyset )$. For a set $Y \subset X_F$, define the map ${\mathfrak {p}}: {\mathfrak {X}} \rightarrow {\mathcal {P}}({\mathfrak {X}})$ as

$$\begin{aligned} {\mathfrak {p}}(Y) := \left\{ (Y \setminus \{y\})^{*rg} \text { for }y \in Y \right\} . \end{aligned}$$

All of the sets contained in ${\mathfrak {p}}(Y)$ are pre-functionally closed RAF sets of chemicals. Moreover, let $X'_F \subset Y$ be a pre-functionally closed RAF set of chemicals which is strictly contained in *Y* and is maximal with this property. Then there is a chemical $y \in Y \setminus X'_F$ and one verifies that $X'_F = (Y \setminus \{y\})^{*rg}$.

Now inductively define the following sets

$$\begin{aligned} \mathfrak {pF}^0&:= \{ X_F^{*g} \} \\ \mathfrak {pF}^{i+1}&:= \bigcup _{Y \in \mathfrak {pF}^i} {\mathfrak {p}}(Y) \text { for all }i \in {\mathbb {Z}}_{\ge 0}. \end{aligned}$$

Due to the finiteness of ${\mathfrak {X}}$, there is a minimal $N \in {\mathbb {N}}$ such that $\mathfrak {pF}^{i+1} = \{ \emptyset \}$ for all $i > N$ (by construction, *N* is bounded by $N \le \vert X_F^{*g} \vert$). The following theorem gives a description of the set of pre-functionally closed RAF sets of chemicals of a CRS, which extends the characterization of the maximal RAF set of chemicals provided in Theorem 4.9.

#### Theorem 4.18

The set

$$\begin{aligned} \mathfrak {pF} := \bigcup _{i=0}^N \mathfrak {pF}^i \end{aligned}$$

is the set of all pre-functionally closed RAF sets of chemicals of the CRS.

#### Proof

By construction, all elements of $\mathfrak {pF}$ are pre-functionally closed RAF sets of chemicals. It remains to show that all pre-functionally closed RAF sets of chemicals are indeed contained in $\mathfrak {pF}$. In this regard, recall that ${\mathfrak {p}}(Y)$ contains all maximal pre-functionally closed RAF sets of chemicals which are strictly contained in *Y*. For a pre-functionally closed RAF set of chemicals $X_F'$, there exists a chain of maximal length of pre-functionally closed RAF sets of chemicals

$$\begin{aligned} X_F' = X_n \subsetneq X_{n-1} \subsetneq \dots \subsetneq X_0 = X_F^{*g}. \end{aligned}$$

Then, $X_F'$ must be contained in $\mathfrak {pF}^n$. $\square$

Finally, the set of functionally closed RAF sets can be obtained by Lemma 4.16 as

$$\begin{aligned} {\mathfrak {F}} = \{ Y^{*g} \text { for } Y \in \mathfrak {pF} \}. \end{aligned}$$

This concludes the application of the semigroup models and their representations by decorated rooted tree to self-generating CRS. The implications of the results are now discussed.

## Discussion

A general discussion of the semigroup models of CRS is given by Loutchko (2022), where, for example, algebraic properties and the possibility to analyze the computational properties of CRS with their semigroup models are expounded upon.

In this article, it was demonstrated how the language of semigroup models provides a natural framework to treat catalytic reaction systems with the RAF property, to determine the maximal RAF set of chemicals and to determine the sets of pre-functionally closed and functionally closed RAF sets of chemicals. The technical basis is provided by the representation of the elements of the semigroup models as decorated rooted trees, because this representation is particularly useful in making the relation of semigroup elements with the F property precise. It will be interesting to investigate whether such representations can be used more generally in the theoretical study of (not necessarily finite) semigroups and semirings. Similar representations have turned out to be useful in the theory of self-similar groups introduced by Nekrashevych (2005).

With regard to CRS theory, the notion of functionally closed sets of RAF chemicals is a very natural concept within the theory of semigroup models. One is naturally led to consider the fixed points of the dynamics, which are RAF sets of chemicals by Proposition 4.7. Moreover, Lemma 4.14 ensures that each RAF set of chemicals has a uniquely determined functional closure with nice properties. The analysis of the set of functionally closed RAF sets of chemicals of a CRS within a living organism can potentially provide insights into the modular organization of its metabolism and the respective control mechanisms. The fact that arbitrary subsets of $X_F$ - in contrast to RAF sets of chemicals - do not have a unique minimal functionally closed RAF set of chemicals which contains them, inspires further investigation of CRS of real biological systems. If a chemical (or a set of chemicals) has a unique minimal functionally closed RAF set of chemicals to which it belongs, then one can conjecture that this particular chemical (or set of chemicals) is specific for the respective functional module. And it is likely that this chemical was acquired together with the respective module in the course of evolution. If, however, this is not the case - such as for the chemical *d* the example shown in Fig. 7, then the respective chemical serves as a kind of mediator between the functional modules in which it is contained. It will be interesting to test such hypotheses on CRS of biological systems and to develop new ones by applying the techniques provided by the semigroup formalism.

Another possibility suggested by the algebraic models of CRS is the coarse-graining obtained by taking quotients of the semigroups which are well-behaved with respect to the algebraic operations. The technical difficulty is thereby to relate the quotients of functions, which live in ${\mathcal {T}}({\mathfrak {X}})$ to quotients of the state space ${\mathfrak {X}}$ in a natural manner. This work is currently being finalized. This more algebraic approach provides an alternative way to reveal and analyze the modularity of a given CRS. Whereas the set of functionally closed RAF sets of chemicals is based on the self-generating property, the quotient structures are not. Therefore, in future, it will be interesting to compare the approach presented in Sect. 4.3 of this article to the algebraic coarse-graining procedures.

## Appendix A List of mathematical symbols

See Table 1.

**Table 1:** List of mathematical symbols used in the article. The first appearance refers to the main text and not to the Mathematical outline

Symbol	Description	Definition/first appearance
(*X*, *R*, *C*, *F*)	A CRS	Definition 2.1
$\text {dom}(r),\text {dom}(R')$	Domain (set of substrates) of a reaction *r* (of a set $R'$ of reactions)	Definition 2.1/Section 2.1
$\text {ran}(r),\text {ran}(R')$	Range (set of products) of a reaction *r* (of a set $R'$ of reactions)	Definition 2.1/Section 2.1
$\pi _R:C \rightarrow R$	Projection $(x,r) \mapsto r$	Section 2.1
$\text {supp}(r),\text {supp}(R')$	Support (set of substrates and products) of a reaction *r* (of a set $R'$ of reactions)	Section 2.1
$X_F$	Set of non-food chemicals	Section 2.1
$R\mid _{X'}$	Set of reactions supported on $X'$	Definition 2.3
$C\mid _{X'}$	Catalysis data supported on $X'$	Definition 2.3
$(X',R\mid _{X'},C\mid _{X'},F)$	subCRS	Definition 2.3
${\mathfrak {X}}$	State space of the CRS	Section 2.2
${\mathcal {P}}(A)$	Power set of a set *A*	Section 2.2
$\phi _r,\phi _x$	Function of the reaction *r*, of a chemical *x*	Section 2.2
${\mathcal {T}}(A)$	Full transformation semigroup of a set *A*	Section 2.2
${\mathcal {S}}$	Semigroup model of the CRS	Definition 2.10
0	Zero function $0 \in {\mathcal {S}}$	Definition 2.10
${\mathcal {S}}^R$	Extended semigroup model of the CRS	Definition 2.10
${\mathcal {S}}(X_F')$	Semigroup model generated by the functions of $X_F'$	Definition 2.12
$\Phi _{X_F'}$	The function of the set $X_F'$	Definition 2.12
${\mathcal {S}}'(X',R\mid _{X'})$	Semigroup model of the subCRS ${(X',R\mid _{X'},C\mid _{X'},F)}$	Remark 2.14
*ext*	Extension of functions ${\mathcal {T}}({\mathcal {P}}(X'_F)) \rightarrow {\mathcal {T}}({\mathfrak {X}})$	Remark 2.14
$T=(V,E,t)$	Rooted tree with vertex set *V* and edge set *E* and root *t*	Section 3
$\text {ch}(v)$	Children of the vertex *v*	Section 3
$T_v$	Subtree rooted at the vertex *v*	Section 3
$\text {lv}(v)$	Level of the vertex *v*	Section 3
$\text {lv}_n(T)$	Set of vertices of level *n*	Section 3
$\text {ht}(T)$	Height of the tree *T*	Section 3
$\text {lf}(T)$	Set of leaves of the tree *T*	Section 3
$T = (A,V,E,t,\omega _V,\omega _E)$	*A*-decorated rooted tree, with $A \subset {\mathcal {T}}({\mathfrak {X}})$	Definition 3.1
${\mathfrak {T}}(A)$	Set of all *A*-decorated rooted trees	Section 3
$T^+ = T_1 + T_2$	Sum of two trees	Definition 3.3
$T^{\circ } = T_1 \circ T_2$	Product of two trees	Definition 3.3
$ev: {\mathfrak {T}}(A) \longrightarrow {\mathcal {T}}({\mathfrak {X}})$	Evaluation map	Definition 3.5
${\mathfrak {T}}$ and ${\mathfrak {T}}^R$	Tree algebras decorated by $\{ \phi _x \}_{x \in X} \cup \{0\}$ and $\{ \phi _r \}_{r \in R} \cup \{0\}$	Sect. 3, after Lemma 3.6
$\tau :{\mathfrak {T}} \longrightarrow {\mathfrak {T}}^R$	Tree algebra homomorphism	Sect. 3, after Lemma 3.6
${\mathcal {D}}^g$	Generative dynamics	Definition 4.6
$Y_0^{*g}$	Fixed point of the generative dynamics	Definition 4.6
$\mathfrak {pF}$	Pre-functionally closed RAF sets of chemicals	Definition 4.12
${\mathfrak {F}}$	Functionally closed RAF sets of chemicals	Definition 4.12
${\mathcal {D}}^{rg}$	Reduced generative dynamics	Section 4.3
$Y_0^{*rg}$	Fixed point of the reduced generative dynamics	Section 4.3
${\mathfrak {p}}: {\mathfrak {X}} \rightarrow {\mathcal {P}}({\mathfrak {X}})$	Map giving maximal pre-functionally closed RAF sets	Section 4.3

## Footnotes

1 In Hordijk and Steel (2017), a subset $R' \subset R$ is called a closed RAF set of reactions if it is RAF and if, in addition, all reactions with a catalyst and support in $\text {supp}(R')$ are elements of $R'$.

2 The F property for a set $R' \subset R$ means that each chemical in $\text {supp}(R') \cap X_F$ satisfies the condition (F) with the $R_i$ satisfying $R_i \subset R'$.

3 Recall that a semigroup ${\mathcal {S}}$ is called *nilpotent* if the ideal ${\mathcal {S}}^n := \{ \phi _1 \circ \phi _2 \circ \cdots \circ \phi _n \vert \phi _i \in {\mathcal {S}} \}$ is trivial, i.e., if ${\mathcal {S}}^n = \{ 0 \}$ for some $n \in {\mathbb {N}}$.

4 A magma is a semigroup without the associativity property.
